# CXCR2 affects sensitization of radioresistant HPV-negative head and neck squamous cell carcinoma cells by ABT-263

**DOI:** 10.1186/s13014-026-02798-w

**Published:** 2026-02-12

**Authors:** Sibylla Kohl, Mareike Schaper, José Arteaga Lajarín, Florentine S. B. Subtil, Rita Engenhart-Cabillic, Ekkehard Dikomey, Sebastian Adeberg, Ulrike Theiß

**Affiliations:** 1 Department of Radiotherapy and Radiation Oncology, Marburg University, Marburg, Germany; 2 Department of Radiotherapy and Radiation Oncology, Marburg University Hospital, Marburg, Germany; 3 National Center for Tumor Diseases (NCT) Frankfurt – Marburg, Marburg, Germany

**Keywords:** HNSCC, HPV negative, Radiosensitization, Senescence, SASP, CXCR2, ABT-263, Senolytics

## Abstract

**Background:**

Radiation-induced senescence strongly contributes to therapy resistance in HPV-negative head and neck squamous cell carcinoma (HNSCC). In particular, the NF-${\rm{\kappa }}$B-dependent arm of the senescence-associated secretory phenotype (SASP) activates signaling pathways that are tightly associated with, and promote, resistance to irradiation. In addition to the SASP, another hallmark of senescence is the upregulation of anti-apoptotic proteins. The BH3-only mimetic ABT-263 has been shown to effectively eliminate senescent cells. In this study, we employed ABT-263 to overcome the therapy-induced resistance in HNSCC cells and uncovered a link to chemokine signaling.

**Methods:**

The HNSCC cell lines Cal33 and UPCI:SCC040 were treated with a combination of ABT-263 and photon irradiation, followed by functional and mechanistic assays assessing viability, apoptosis, senescence, secreted proteins, clonogenic survival, and DNA repair.

**Results:**

Functionally, ABT-263 induced apoptosis via impeding Bcl-xL and activating Bax. Senescence levels were reduced after irradiation. Mechanistically, we observed cell-line- and protein-specific changes in the SASP, including a striking difference in CXCR2 receptor expression. Cal33 cells exhibited strong downregulation of CXCR2 and were radiosensitized by ABT-263, as indicated by reduced viability and clonogenic survival. In contrast, CXCR2 expression was induced in UPCI:SCC040 cells following treatment; although viability was diminished, clonogenic survival remained unaffected. Notably, radiosensitization in UPCI:SCC040 cells could be achieved through concomitant inhibition of CXCR2. Furthermore, the radiosensitizing effect was not attributable to increased DNA damage, as evidenced by $\gamma$H2AX/53BP1 co-localization.

**Conclusions:**

These findings highlight a central role for CXCR2-mediated signaling in the development of radioresistance in HPV-negative HNSCC cells.

**Clinical trial number:**

Not applicable

**Supplementary Information:**

The online version contains supplementary material available at 10.1186/s13014-026-02798-w.

## Background

Radiotherapy is a central component of treatment for HPV-negative head and neck squamous cell carcinoma (HNSCC) and can be administered at all tumor stages as part of standard of care [1]. Despite intensive treatment regimens delivering cumulative doses of 60–80 Gy, local relapse frequently occurs within three years after first-line therapy [2, 3]. Such high recurrence rates illustrate the major clinical challenge in managing locally advanced HNSCC, as recurrent disease is often associated with poor functional outcomes, limited curative salvage options, and markedly reduced overall survival. These unmet needs highlight the urgency of developing strategies that improve durable tumor control in this patient subgroup. The causes of therapy resistance and recurrence are multifactorial and include environmental influences, tumor heterogeneity, cellular plasticity, and dormancy, amongst other factors [4–6].

Radiotherapy influences tumor cell fate in several ways, and one process that significantly contributes to disease relapse is therapy-induced senescence [7, 8]. Senescence is a cellular stress response commonly triggered by tumor therapy. After irradiation, DNA damage activates sensing proteins such as ATM, initiating the DNA repair machinery to preserve genomic integrity. A persistent DNA damage response (DDR), together with irreparable genomic damage is characteristic of cells undergoing senescence [9, 10]. Senescent cells typically display stable growth arrest and extensive metabolic alterations. A hallmark of senescence is the robust production of a complex mixture of secreted factors known as the senescence-associated secretory phenotype (SASP), which drives non-cell-autonomous effects within the microenvironment [11]. The composition of the SASP is highly context-dependent and varies with mode of induction, cell type, spatial distribution, and time point examined. It includes pro-inflammatory cytokines and chemokines, matrix metalloproteases, growth factors, and insoluble components such as collagens. Their distribution within the tumor microenvironment can activate pro-proliferative and pro-tumorigenic signaling pathways, ultimately promoting tumor regrowth [12].

We recently demonstrated that radiation-induced senescence in HNSCC cells is a strong indicator of radioresistance [13], with both senescence and SASP levels correlating positively with the degree of resistance. The secretion of NF-${\rm{\kappa }}$B-dependent inflammatory SASP factors suggests a paracrine mechanism of resistance, and the chemokine receptor CXCR2 and its ligands show prognostic relevance [14]. The context-specific composition of the SASP appears to support survival, proliferation, and regeneration in treatment-resistant cells. This opens opportunities for therapeutic intervention, as senomorphic agents such as metformin can suppress the SASP, thereby sensitizing cells to irradiation or modulating their immunophenotype [14–16]. These findings provide a rational for emerging clinical trials evaluating senomorphics in combination with radiochemotherapy [17, 18].

Another class of senotherapeutics –senolytics – selectively eliminates senescent cells [16]. Senolytics have distinct advantages over senomorphics: whereas senomorphics suppress the SASP and require continuous administration to maintain this effect, senolytics eradicate the SASP source itself—the senescent cell—allowing treatment discontinuation once clearance is achieved [19]. Senescent cells depend on resistance to both extrinsic and intrinsic apoptosis to maintain their arrested state. Senolytics exert cytotoxic effect by targeting these anti-apoptotic adaptations, often mediated by upregulation of Bcl-2 family proteins including Bcl-w, Bcl-xL, Bcl-2 and Mcl-1 [20].

ABT-263 is a well-established Bcl-2/Bcl-xL-inhibitor. As a BH3-only mimetic, it binds with high affinity to the BH3 domains of Bcl-2 family members, thereby preventing them from inhibiting pro-apoptotic proteins such as Bax and Bad. This promotes pore formation in the mitochondrial outer membrane, enabling cytochrome c release and subsequent activation of the caspase cascade, ultimately triggering intrinsic apoptosis [21, 22]. Preclinical studies in HNSCC cell lines and mouse models have shown enhanced anticancer activity when Bcl-2 inhibitors are combined with chemotherapy or radiotherapy, although their effects as monotherapies remain inconclusive [23]. Treatment-induced senescence can be successfully abrogated by ABT-263 in vitro [24]. In vivo, ABT-263 prolongs time to relapse and reduces fibrosis, a frequent normal tissue complication after radiotherapy [25]. To date, approximately 60 clinical trials have investigated Bcl-2-inhibitors as monotherapies or in combination with other anticancer agents for solid tumors, but data on their use with radiotherapy are not yet available [26].

We examined the impact of combining radiotherapy with ABT-263 on in-vitro cell survival, with the aim of identifying biomarkers that may enhance therapeutic strategies for the subset of radioresistant HNSCC. Following assessment of drug-induced cytotoxicity, cellular viability was quantified in two HPV-negative HNSCC cell lines, UPCI:SCC040 and Cal33, both of which exhibit high radioresistance and elevated senescence levels [14]. Radiosensitization by ABT-263 was evaluated through clonogenic survival assays after combined treatment. Functionally, the impact of ABT-263 was tested by analyzing apoptosis, senescence, and the SASP. Our results indicate that ABT-263 combined with irradiation reduces cell viability and effectively radiosensitizes Cal33 cells by promoting apoptosis and diminishing irradiation-induced senescence. Mechanistically, in UPCI:SCC040 cells, additional knockdown of CXCR2 –a key receptor for senescence-associated cytokines – was required to achieve radiosensitization.

In conclusion, the anti-apoptotic Bcl-2 signaling pathway represents a promising therapeutic target in radioresistant HNSCC, while CXCR2 expression may serve as a predictor of therapeutic efficacy. Further investigation into the interplay between these pathways is warranted.

## Materials and methods

### Cell lines and cell culture

HPV-negative HNSCC cell lines UPCI:SCC040 and Cal33 were purchased from DSMZ (RRID:CVCL_2222 and CVCL 1108, DSMZ, Germany Cat#ACC660 and Cat#ACC447). Cell line identity was confirmed by STR profiling and comparison with the Expasy and DSMZ databases. Cells were cultured in DMEM (Thermo Fisher Scientific Inc. Cat#12077549) supplemented with 10% FBS (Capricorn Scientific GmbH Cat#FBS-11A) and 1% penicillin/streptomycin (Sigma-Aldrich Cat#P0781), and maintained at 37 °C in a humidified atmosphere containing 7.5% CO_2_. Routine PCR-based tests consistently confirmed the absence of mycoplasma contamination. Cell numbers used for each assay are provided in Supplementary Table 1.

### Treatment of cells

**ABT-263 (Navitoclax) treatment**: Unless stated otherwise, cells were treated with ABT-263 (BIOZOL Cat#APE-A3007, CAS No0.923564-51–6) at a working concentration of 5 µM for the UPCI:SCC040 cell line and 1 µM ABT-263 for the Cal33 cell line. The drug was applied immediately before irradiation and remained in the medium until end of the experiment. Control samples received an equivalent volume of DMSO.

**X-Ray Irradiation**: Cells were irradiated using the Precision X-RAD 320ix biological irradiator (Precision X-Ray, North Branford, CT, USA, RRID: SCR026275) located at the Core Facility of Marburg University. Irradiation was performed at 320 kV and 8 mA with a dose rate of 1.1 Gy/min, and using a Thoraeus filter (0.5 mm Cu+ 0.5 mm Al). Preliminary dosimetry confirmed the absolute dose delivered. Doses of 1, 2, 4 and 6 Gy were applied as indicated.

**siRNA transfection**: Transient knockdown of CXCR2 was performed by siRNA transfection as previously described [13]. Lipofectamine 2000 (Invitrogen Cat#11668019) was used according to the manufacturer’s instructions together with a pool of four CXCR2-specific oligonucleotides or a non-targeting control siRNA pool (Dharmacon, Horizon Discovery Group; sequences in Supplementary Table 2). Cells were used for further experiments 24 h post-transfection. Knockdown (KD) efficiency was confirmed by qRT-PCR using samples which were harvested 24 h after transfection.

### Viability assay

Viability was determined using the alamarBlue™ Cell Viability Reagent (Thermo Fisher Scientific Inc. Cat#10099022) following the manufacturer´s protocol. Viability was measured on day 6 after irradiation. Substrate conversion was quantified using a Varioskan LUX microplate reader (Thermo Fisher Scientific Inc., RRID: SCR026792) at 560 nm excitation and 590 nm emission. Blank values were subtracted and results normalized to control samples.

### Flow cytometry

The flow cytometry was performed on a CytoFlex-Lx (Beckman-Coulter, Brea, CA, USA, RRID: SCR025067) at the FACS Core Facility, of Marburg University. Data were analyzed using FlowJo v10 Software (RRID:SCT_008520, Tree star Inc.; Ashland, OR, USA). For all assays, the gating strategy involved exclusion of debris, removal of doublets, and quantification of fluorescence signals (Suppl. Fig. 1).

**Annexin V assay**: Viable, apoptotic and necrotic cells were distinguished based on membrane phosphatidylserine exposure and DNA staining using FITC-Annexin V and propidium iodide (PI). Cells (5–10 ×10^4^ cells/cm^2^) were seeded and treated after 4 h adherence period. Measurements were performed on day 4 post- irradiation. The Annexin V-FITC Detection Kit (PromoCell GmbH Cat#PK-CA577–K101-100) was used according to the manufacturer’s protocol. Cells negative for both FITC and PI (FITC-/PI-) were considered viable; FITC+/PI – cells were classified as early apoptotic; FITC+/PI+ cells as late apoptotic or necrotic (Suppl. Fig. 1A).

**Senescence assay**: Senescence-associated β-galactosidase (SA-β-gal) activity was measured as described [27]. Cells (16 - 27 × 10^4^ cells/cm^2^) were seeded, treated as indicated, and incubated for 4 days. SA-β-gal activity was visualized by 1 h incubation with 50 µM C12FDG (Abcam Cat#273642, CAS no.138777-25–0). Lysosomal alkalization was induced with 100 nM bafilomycin A1 (Bio-Techne GmbH Cat#1334, CAS no.88899–55-2) for 1 h to ensure lysosomal origin of the signal. Cells with high C12-fluorescein and high SSC were classified as senescent (Suppl. Fig. 1B).

### qRTPCR

Total RNA was extracted from scraped cell pellets using NucleoSpin RNA II Kit (Macherey-Nagel Cat#740955.50). cDNA was synthesized using the High-Capacity cDNA Reverse Transcription Kit (Thermo Fisher Scientific Inc. Cat#10400745) with 1 µg RNA, 4 mM dNTPs, 50 U MultiScribe™ Reverse Transcriptase and 1x random primers. qRT-PCR was performed on a QuantStudio5 Real-Time PCR System (Thermo Fisher Scientific Inc., RRID: SCR020240). Relative gene expression was determined using the ΔΔCT method, with ALAS as the reference gene. Primer sequences are listed in Supplementary Table 3.

### Protein detection

**Western Blot**: Western Blotting was performed as described in [28]. Cells in exponential growth were harvested, and protein detection was carried out using antibodies listed in Supplementary Table 4. Bands were visualized using an ECL chemiluminescence detection system (Bio-Rad, Feldkirchen, Germany).

**ELISA**: Cell-free supernatants were collected for quantification of secreted proteins. Cytokines IL-1α, IL-1β, IL-8 and CXCL1 were measured using DuoSet ELISA kits (R&D Systems, IL-1α Cat#DY200, IL-1β Cat#DY201-05, IL8 Cat#30021151, CXCL1 Cat#DY 275) according to manufacturer instructions. Cytokine levels were normalized to the ratio of seeded cell number to the number of cells counted at harvest.

### Clonogenic survival assay

Cells in exponential growth were pre-plated at densities of 10–250 cells/cm^2^. After 10–14 days, colonies ( > 50 cells) were fixed and stained (4% paraformaldehyde, 0.1% crystal violet). Plating efficiency was calculated, and survival was normalized to untreated controls. Survival curves were fitted using the linear-quadratic model via least-squares regression. The D_10_ (dose at 10% survival) and D_37_ values were determined from the fitted curve.

### DSB repair detection

DSB repair was assessed by co-staining γH2AX and 53BP1 as described in [29]. Cells were fixed 24 h after irradiation to quantify residual DSBs. Primary antibodies used were mouse anti-phospho-S139–H2AX (1:500; Millipore Cat#05–636, RRID:AB_309864) and rabbit anti-53BP1 (1:500, Novus Biologicals Cat#NB100-305, RRID:AB_10001695). Secondary antibodies were AlexaFluorPlus647 goat anti-mouse IgG (1:1000; Invitrogen Cat#A-32728, RRID:AB_2866490) and AlexaFluor488 donkey anti-rabbit IgG (1:1000; Invitrogen Cat#A-21206, RRID:AB_2535792). Slides were mounted with ProLong Gold antifade containing DAPI (Invitrogen, Cat#11539306). Imaging was performed on an Olympus I×83 microscope (Olympus Europa Holding GmbH, RRID: SCR020344) at 60x magnification. Approximately 12 z-stacks (0.35 µm) were captured and maximally projected across the z-plane. For each condition, 100 nuclei were manually counted.

### Statistics and data analysis

All experiments were carried out in biological triplicates unless stated otherwise. Data are represented as mean ± standard error of the mean (SEM). Statistical significance was assessed using unpaired Student’s t-test. Values of *p* < 0.05 were considered significant. The Combination Index (CI) was calculated to assess the effect of irradiation combined with ABT-263 treatment [30]. For the Bliss Independence model, the CI was defined as CI = (E_ABT-263_ + E_irradiation_ - E_ABT-263_ * E_irradiation_)/E_combined treatment_, where E represents the measured effect. CI values below 1 indicate synergistic interactions. The Highest Single Agent (HSA) reference model was calculated as CI = max (E_ABT-263_, E_irradiation_)/E_combined treatment_,. In this model, positive values indicate cooperative effects, with increasing value reflecting stronger cooperation. Data analysis was conducted using Microsoft Excel and GraphPad Prism software (GraphPad Software Inc., RRID:SCR_002798).

## Results

### ABT-263 induces apoptotic cell death in HPV-negative cells

**Fig. 1:**
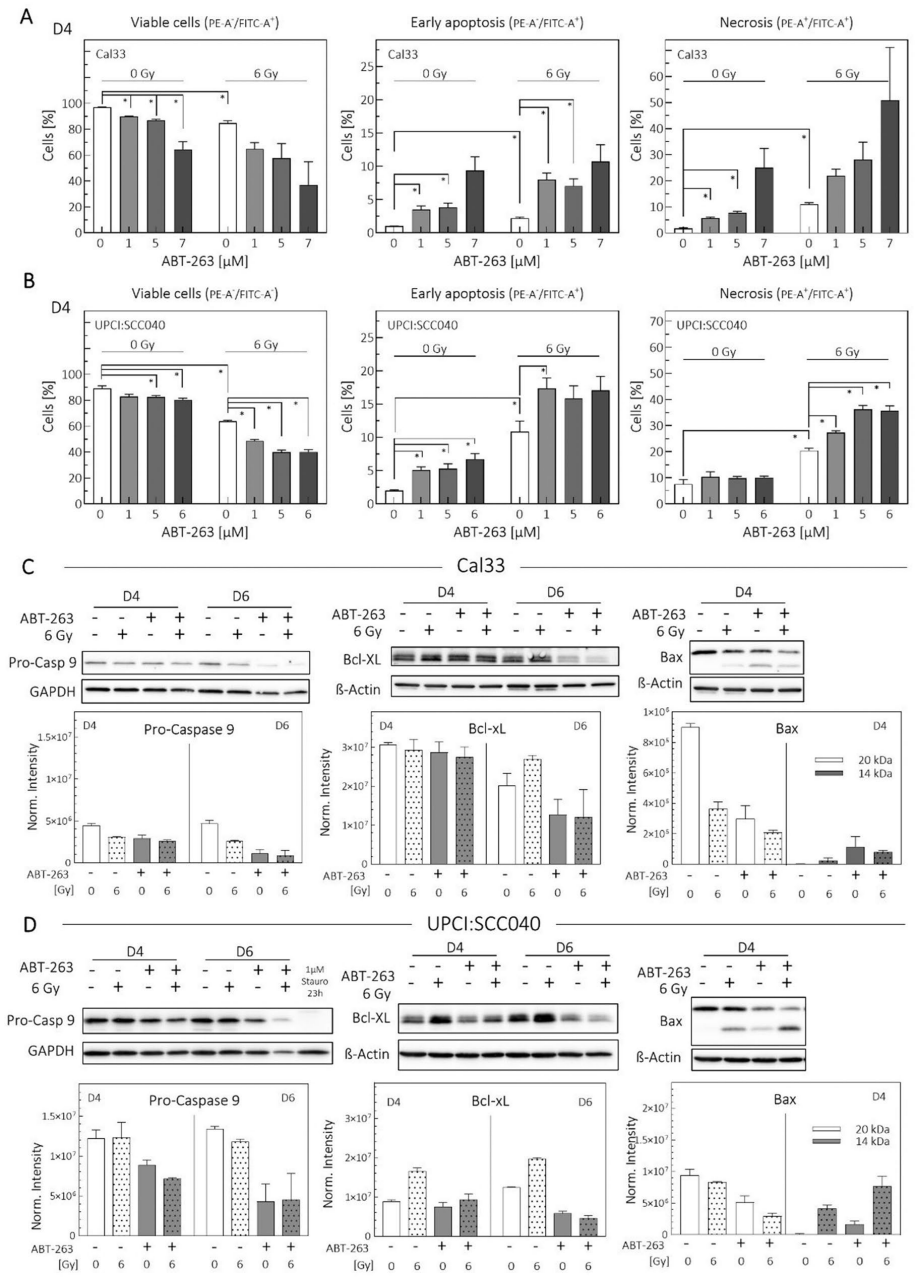
ABT-263 induces cell death by inhibiting anti-apoptotic and increasing pro-apoptotic proteins. Cells were treated with 1–7 µM ABT-263 and 6 Gy irradiation. (**A**) Cal33 (*N* = 2) and (**B**) UPCI:SCC040 (*N* = 4). AnnexinV assays were performed to assess viable, early apoptotic and late apoptotic/necrotic cells 4 days after treatment. Values represent means ± SEM of N replicates. Statistical significance was determined using Student’s t test, *p* < 0.05 (*). (**C**) Cal33 and (**D**) UPCI:SCC040 protein lysates were analyzed 4 (D4) or 6 (D6) days after treatment. Representative Western blots and densitometric quantification (*N* = 2) are shown for pro-Caspase-9, Bcl-xL and Bax. Stauro = staurosporine

To examine the effects of ABT-263, we used Western Blot analysis and Annexin V assays to assess apoptosis induction (Fig. 1, Suppl. Fig. 1A). The examined cell lines, Cal33 and UPCI:SCC040, are HPV-negative squamous cell carcinoma line, with well characterized responses to irradiation [31, 32]. In Cal33 cells, ABT-263 monotreatment decreased viability and increased early apoptosis and necrosis (Fig. 1A). The effect was significant at 1 µM and continued to increase with higher concentrations. When combined with irradiation, apoptosis and necrosis levels were higher than in either treatment alone. UPCI:SCC040 cells also showed a significant reduction in viability when treated with ABT-263 alone at concentrations ≥ 5 µM (Fig. 1B). Increasing concentrations elevated early apoptosis, but necrosis did not increase. Combined treatment significantly enhanced both apoptosis and necrosis, suggesting a sensitizing effect. Calculation of the combination index (CI) according to the Bliss Independence model [30] indicated synergistic induction of necrosis in Cal33 cells at all drug concentrations and in UPCI:SCC040 cells at higher concentrations. Effects on early apoptosis remained additive (Supplementary Table 5).

Western Blot analysis confirmed apoptosis induction through increased pro-apoptotic protein levels and inhibition of anti-apoptotic proteins. Pro-Caspase 9, the inactive form of Caspase 9, was cleaved in response to apoptotic stimuli. In irradiated Cal33 cells, protein levels slightly decreased, becoming more pronounced on day 6 and in combination with ABT-263 (Fig. 1C and Suppl. Fig. 2A). Bcl-xL, a potent apoptosis inhibitor, increased after irradiation on day 6, consistent with senescence induction [14]. ABT-263 reduced Bcl-xL levels. Bcl-xL acts inhibitory on Bax by preventing its mitochondrial assembly. Bax (Fig. 1C and Suppl. Fig. 2A, C) remained inactive in untreated samples appearing at its cytosolic 20 kDa form. A degraded 14 kDa band, representing the monomer bound to mitochondria [33], indicated Bax activation, which became detectable after ABT-263 and irradiation.

In UPCI:SCC040 cells, Pro-Caspase 9 decreased in response to ABT-263, with an additional reduction after irradiation detectable on day 4 (Fig. 1D and Suppl. Fig. 2B). Bcl-xL levels increased after irradiation on day 4, and ABT-263 suppressed this effect (Fig. 1D and Suppl. Fig. 2B). Bax activation, indicated by the 14 kDa band, was strongest in the combined treatment (Fig. 1D and Suppl. Fig. 2B, C).

In both HNSCC cell lines, ABT-263 clearly induced apoptosis by activating the pro-apoptotic signaling cascade. The drug had particularly strong effects in irradiated samples, potentially due to increased apoptosis in irradiation-induced senescent cells or in cells with elevated Bcl-xL.

### ABT-263 diminishes irradiation-induced senescence

**Fig. 2:**
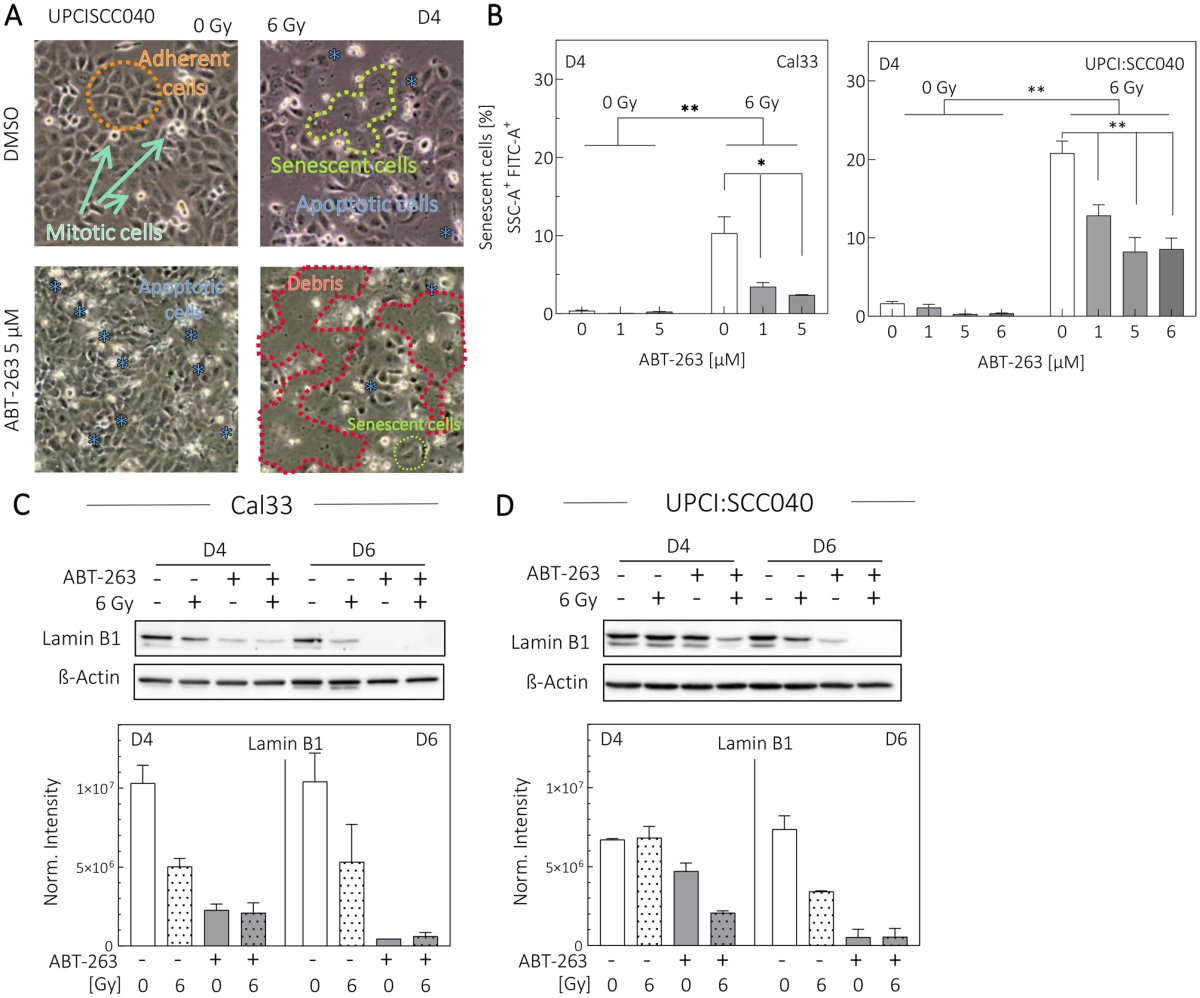
ABT-263 decreases senescence in Cal33 and UPCI:SCC040 cells. Cells were treated with 1–6 µM ABT-263 and 6 Gy irradiation and analyzed on day 4 (D4) or day 6 (D6). (**A**) Representative images of UPCI:SCC040 cells treated with 5 µM ABT-263, 6 Gy irradiation, and controls. Morphology is shown for adherent viable cells (orange), proliferating mitotic cells (green), apoptotic cells (blue), senescent cells (yellow) and degraded debris (red). (**B**) Flow-cytometric analysis of senescence-associated β-galactosidase–positive cells in Cal33 (left panel) and UPCI:SCC040 (right panel). Values represent means ± SEM of *N* ≥ 3 replicates. Student´s t test: *p* < 0.05 (*), *p* < 0.01 (**). (**C-D**) Lamin B1 detection by Western Blot with densitometric quantification (*N* = 2)

We next examined whether ABT-263 affects the treatment-induced senescent. The cells exhibited distinct morphological changes under the different conditions. While unirradiated cells remained mitotically active, ABT-263 treatment increased apoptotic morphology (Fig. 2A, panels to the left). Irradiation alone induced both apoptosis and senescence, the latter characterized by enlarged cell morphology. Combined treatment produced ruptures in the monolayer, leaving mostly cell debris and fewer visible senescent cells (Fig. 2A, panels rightward).

Detection of senescence-associated ${\rm{\beta }}$-galactosidase (Fig. 2B, Suppl. Fig. 1B) indicated minimal senescence in untreated controls (0.37 - 1.63%). Four days after 6 Gy irradiation, senescent cells increased to 10.3% in Cal33 and 20.79% in UPCI:SCC040 cells. ABT-263 diminished this population 2–3-fold in a dose-dependent manner (Fig. 2B). Loss of Lamin B1, a senescence marker [34], occurred in irradiated samples of both cell lines (Fig. 2C,D). Because Lamin B1 is a Caspase 6 substrate [35]. its further reduction after ABT-263 treatment may also reflect apoptosis.

Overall, morphological changes and reduced ${\rm{\beta }}$-galactosidase activity indicate that ABT-263 diminishes the senescent cell population in both cell lines.

### ABT-263 sensitizes Cal33, but not UPCI:SCC040 cells to irradiation

The pro-apoptotic and senolytic activities of ABT-263 confirmed its cell-killing capabilities. We next assessed whether these effects sensitized cells to irradiation using Alamar Blue viability assays and clonogenic survival assays.

**Fig. 3:**
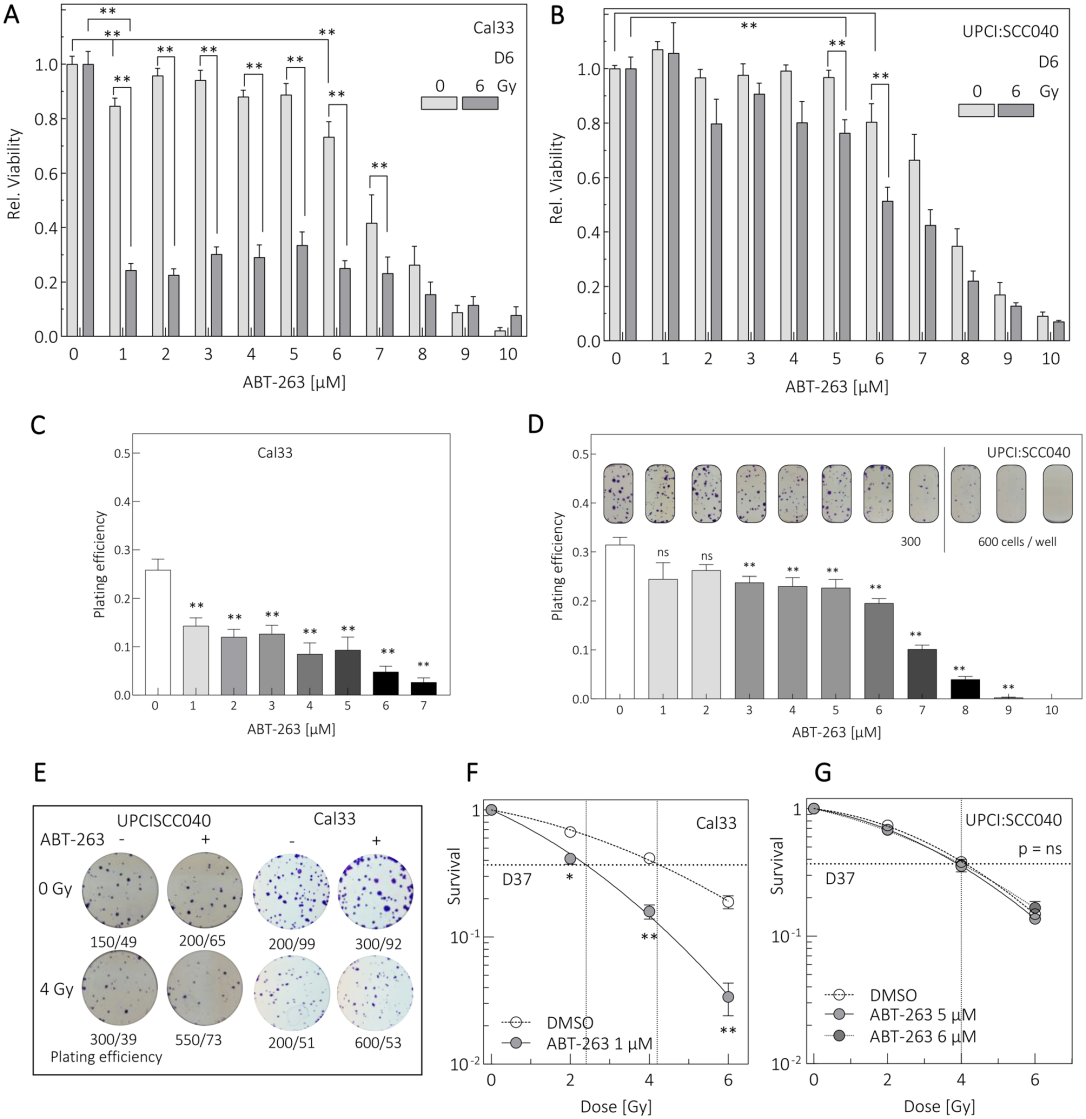
ABT-263 affects viability and clonogenic survival of HPV-negative HNSCC cells. Alamar blue assays were performed on day 6 (D6) after treatment with 6 Gy irradiation and various ABT-263 concentrations for (**A**) UPCI:SCC040 and (**B**) Cal33. Values were normalized to DMSO controls. Plating efficiency was assessed via colony formation assays in (**C**) Cal33 and (**D**) UPCI:SCC040 cells treated with ABT-263. (**E**) Representative colony formation images after irradiation and ABT-263 treatment. (**F,G**) Colony formation assay after 2, 4 and 6 Gy irradiation. (**F**) Cal33 treated with 1 µM ABT-263. (**G**) UPCI:SCC040 treated with 5 or 6 µM ABT-263. DMSO served as control. Values represent means ± SEM of *N* ≥ 3 replicates. Student´s t test: *p* < 0.05 (*), *p* < 0.01 (**), ns: not significant

Viability assays used ABT-263 concentrations of 1–10 µM with 6 Gy irradiation; controls were untreated. To visualize radiosensitizing effects, irradiated samples were normalized to match unirradiated controls (Fig. 3A,B 0 µM bars). ABT-263 monotherapy reduced viability in both lines, with significant reductions at ≥ 6 µM (Fig. 3A,B light grey bars). Cal33 cells showed a non-significant trend toward reduced viability at lower concentrations. In combination with irradiation, Cal33 cells were highly sensitive to ABT-263 (Fig. 3A, dark grey bars). Instead of the additive pattern expected from normalized data, viability declined sharply, indicating significant profound radiosensitization at 1–7 µM. At higher concentrations, drug cytotoxicity dominated. In UPCI:SCC040 cells, ABT-263 monotherapy significantly reduced viability at ≥ 6 µM (Fig. 3B, light grey bars). Combined treatment reduced viability significantly, but radio-sensitization was only evident above 5 µM (Fig. 3B, dark grey bars). The optimal therapeutic window was 5–6 µM. At higher concentrations, cytotoxicity dominated; at lower concentrations, no clear effect occurred.

ABT-263 also reduces clonogenic survival. In Cal33 cells, plating efficiency decreased significantly at 1 µM, and colonies were absent at ≥ 7 µM (Fig. 3C). In UPCI:SCC040, plating efficiency decreased significantly at ≥ 3 µM with no colonies at 10 µM (Fig. 3D). Due to the strong effect in Cal33 cells, 1 µM ABT-263 was used for combined colony formation assays (Fig. 3E). After normalization, ABT-263 produced pronounced radiosensitization (D37_control_: 4.1 Gy versus D37_ABT-263_: 2.2 Gy; Fig. 3 F). In UPCI:SCC040, 5 and 6 µM ABT-263 were tested (Fig. 3G); neither concentration radiosensitized the cells (D37: 4 Gy for all conditions).

The Highest Single Agent model was used to calculate the CI (Supplementary table 6) for the dual treatment, indicating a highly cooperative effect in Cal33 cells when ABT-263 is combined with irradiation. This effect was considerably smaller for UPCI:SCC040.

ABT-263 strongly impaired proliferation and survival in Cal33 cells and acted as a potent radiosensitizer. Surprisingly, UPCI:SCC040 cells did not respond similarly, despite activation of apoptosis and senescence reduction in both lines.

### ABT-263 alters gene expression and secretion of SASP factors

Previous work demonstrated that irradiation-induced senescence is accompanied by secretion of NF-κB-dependent cytokines that promote survival and proliferation in nearby cells [13, 14, 31]. We hypothesized that cytokine dependency contributes to the differential responses of the two cell lines to ABT-263. We therefore analyzed a panel of cytokines and chemokines at both gene expression and protein secretion level.

**Fig. 4:**
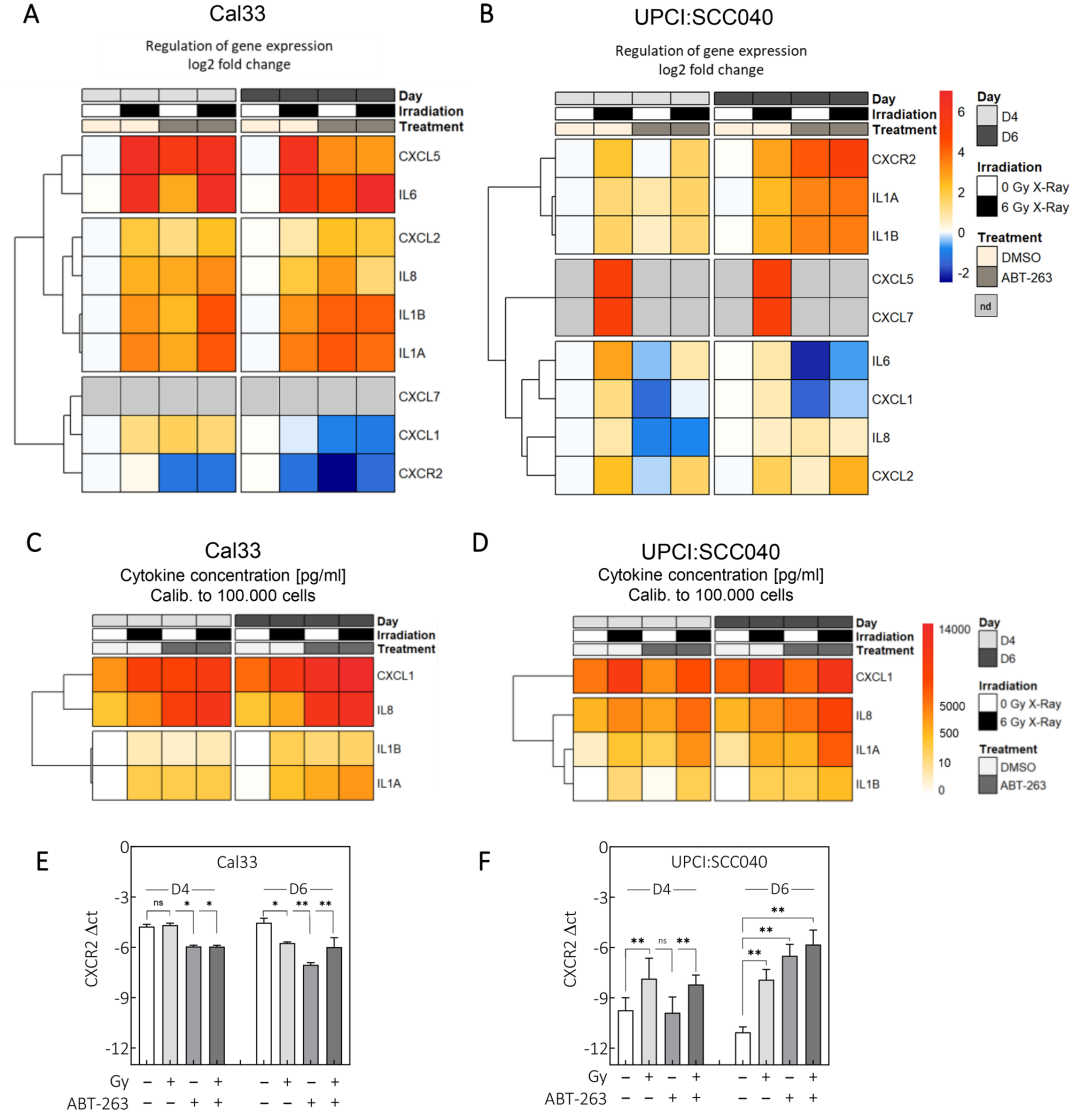
ABT-263 decreases senescence and alters the SASP in UPCI:SCC040 and Cal33 cells. Cells were treated with 1 µM ABT-263 (Cal33) or 5 µM of ABT-263 (UPCI:SCC040) concomitantly with 6 Gy irradiation and analyzed on D4 or D6. (**A-B**) Gene expression of secreted factors measured by qRT-PCR and normalized to unirradiated DMSO controls. (**A**) UPCI:SCC040. (**B**) Cal33. (**C-D**) Secreted IL1A, IL1B, IL8 and CXCL1 protein levels were detected by ELISA on D4 and D6. Protein concentrations (pg/ml) were normalized to cell number. (**C**) UPCI:SCC040. (**D**) Cal33. (**E-F**) Relative CXCR2 gene expression shown as ΔCT values in (**E**) Cal33 and (**F**) UPCI:SCC040 cells. Values a represent means ± SEM of *N* = 2. Student’s t test: *p* < 0.05 (*), *p* < 0.01 (**), ns: not significant

Apart from minor differences in secretion profiles, no factor explained the observed functional differences. However, because many chemokines signal through CXCR2, we examined CXCR2 expression and found distinct treatment-related alterations (Fig. 4, Suppl. Fig. 3 and 4, Supplementary Table 7). For Cal33 cells, most examined factors were upregulated four days after irradiation (Fig. 4A, Suppl. Fig. 3A-D,I-K). CXCL5 and IL6 showed strong and sustained upregulation. CXCL2, IL8, IL1B and IL1A were also consistently elevated. ABT-263 did not reduce their expression. In contrast, CXCL1 and CXCR2 were downregulated by irradiation on day 6, and further suppressed by ABT-263. CXCL7 was undetectable. In UPCI:SCC040 cells, all nine genes were upregulated after irradiation (Fig. 4B, Suppl. Fig. 3E-H,L-O). CXCR2, IL1A and IL1B were especially elevated, including after ABT-263. CXCL5 and CXCL7 were undetectable in untreated cells but increased after irradiation and were suppressed again by ABT-263. ABT-263 downregulated IL6 and CXCL1 expression and inhibited irradiation-induced expression. CXCL2 and IL8 were minimally affected. Protein secretion patterns mirrored gene expression (Fig. 4C,D, Suppl. Fig. 4A,B). ABT-263 alone increased secretion, and combined treatment acted additively for IL-1a and IL-1b in UPCI:SCC040, while levels were markedly lower in Cal33. Irradiation-induced IL8 secretion was further increased by ABT-263 in both cell lines, whereas CXCL1 secretion decreased.

CXCR2 expression was regulated in opposite directions: irradiation and ABT-263 decreased it in Cal33 but increased it in UPCI:SCC040 (Fig. 4E,F, Suppl. Fig. 3P,Q). In Cal33, irradiation decreased CXCR2 expression at day 6 and ABT-263 treatment has a similar effect. By contrast, receptor expression was enhanced in UPCI:SCC040 cells after irradiation, ABT-263 and in combination on day 6.

In summary, ABT-263 affected SASP factor expression in both cell lines, but the most striking difference was CXCR2 regulation: reduced in Cal33 and increased in UPCI:SCC040.

### Inhibition of CXCR2 signaling triggers ABT-263-induced radiosensitization in UPCI:SCC040 cells

**Fig. 5:**
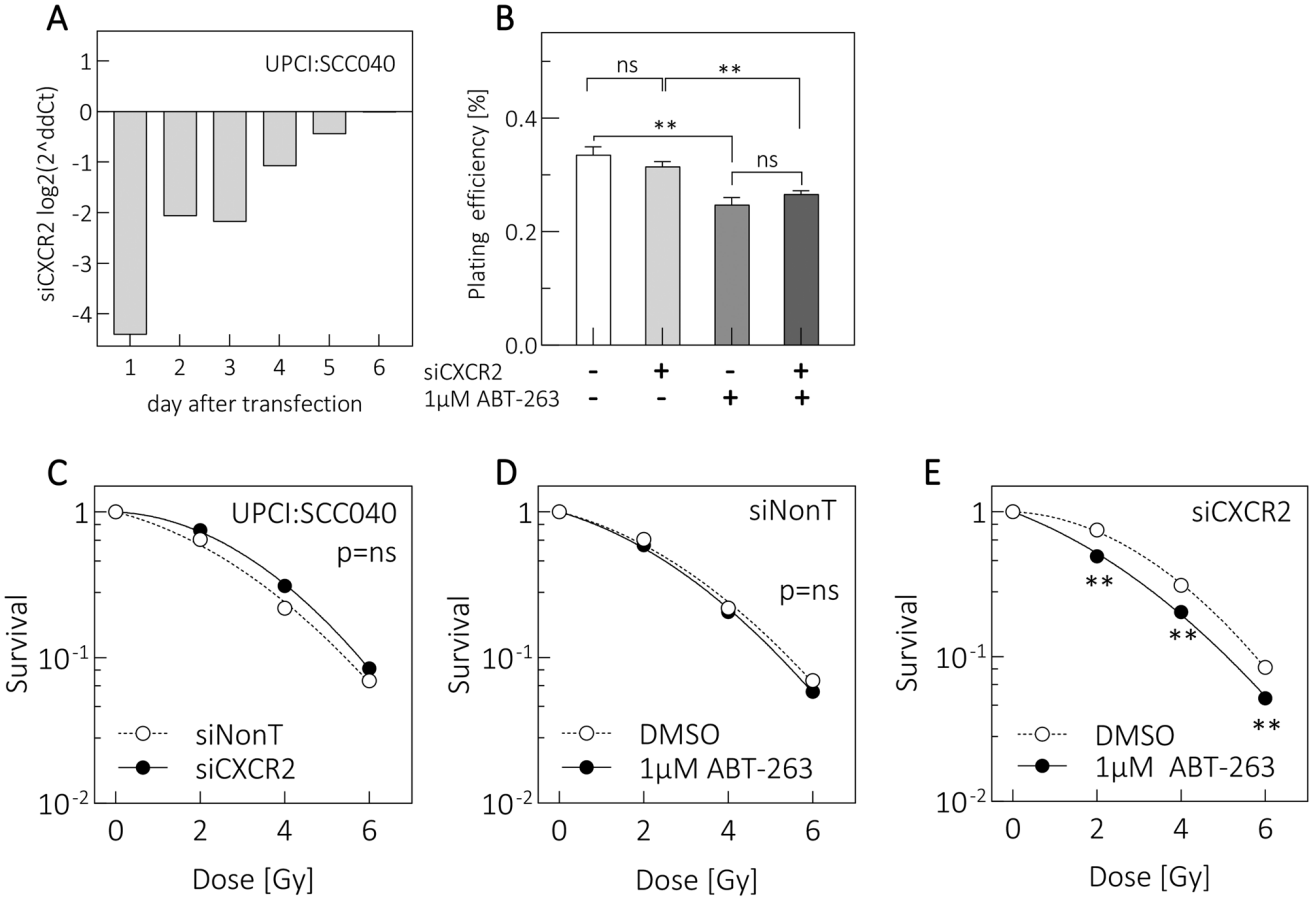
CXCR2 knockdown (KD) in combination with ABT-263 treatment radiosensitizes UPCI:SCC040 cells. (**A**) Time-course analysis of CXCR2 KD, validated by qRT-PCR 6 days after transfection. Expression was normalized to the siNont control. (**B**) Plating efficiency was assessed by colony formation. Cells were reseeded 24 h after transfection and treated with 1 µM ABT-263 after adhesion. DMSO served as control. (**C-D**) Colony formation after 2, 4 and 6 Gy irradiation. (**C**) Survival curves of untreated UPCI:SCC040 siNont vs. siCXCR2 KD. (**D-E**) Survival curves of (**D**) siNont and (**E**) siCXCR2 cells with 1 µM ABT-263 immediately prior to irradiation. Values are are means ± SEM of *N* ≥ 3 replicates. Student’s t test: *p* < 0.05 (*), *p* < 0.01 (**), ns: not significant

To test whether CXCR2 contributes to therapy resistance, we inhibited CXCR2 expression in UPCI:SCC040 cells using sequence-specific siRNA (siCXCR2) or a non-targeting control (siNonT). Knockdown persisted for at least three days (Fig. 5A) and plating efficiency was unaffected (Fig. 5B). Clonogenic survival after irradiation alone did not differ between siNonT and siCXCR2 cells (Fig. 5C). In siNonT cells, ABT-263 did not alter clonogenic survival (Fig. 5D). Remarkably, siCXCR2 cells became significantly radiosensitized after ABT-263 administration (Fig. 5E). The results support the hypothesis that CXCR2 signaling contributes to radioresistance.

### Radiosensitization via ABT-263 treatment is not triggered by DNA damage

**Fig. 6:**
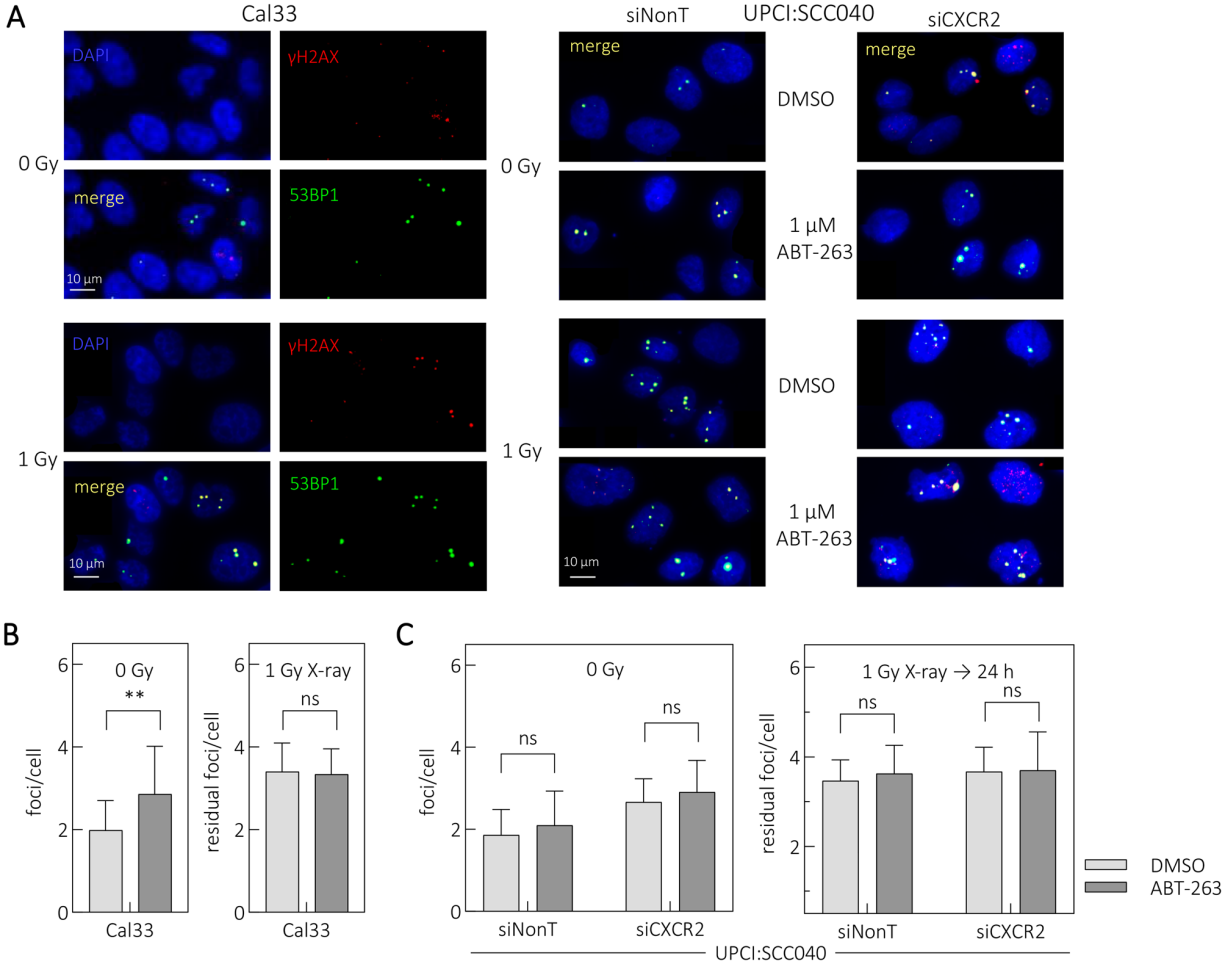
siCXCR2 KD does not alter DSB repair foci after ABT-263 treatment and 1 Gy irradiation. (**A**) Representative images of DSBs in Cal33 (left) and UPCI:SCC040 (right). Nuclei were stained with DAPI (blue); residual DSBs at 24 h post-irradiation were visualized by γH2AX (red) and 53BP1 (green). (**B**) Residual DSB foci in Cal33 cells treated with 1 µM ABT-263 before irradiation. DMSO served as control. (**C**) Residual DSB foci in transfected UPCI:SCC040 cells treated with 1 µM ABT-263 before irradiation, comparing siCXCR2 with siNont. Values represent means ± SEM of *N* ≥ 3 replicates, background foci were subtracted. Student’s t test: *p* < 0.05 (*), *p* < 0.01 (**), ns: not significant

To determine whether ABT-263 alters DNA damage responses, we examined residual DNA damage foci in Cal33 cells and in UPCI:SCC040 cells transfected with siNonT or siCXCR2. The cells were treated with 1 Gy irradiation and 1 µM ABT-263 (Fig. 6A). In Cal33 cells, ABT-263 alone increased residual foci, but combined treatment did not alter residual foci compared to irradiation alone (Fig. 6B). UPCI:SCC040 cells showed no differences in residual foci between treatments, regardless of siRNA condition (Fig. 6C).

Thus, ABT-263-induced radiosensitization was not associated with increased residual DNA damage, supporting the hypothesis that radiosensitization results from changes in secretome-related signaling.

## Discussion

In this study, we aimed to identify a druggable biomarker that could improve current treatment strategies for radioresistant, locally advanced HNSCC. Senescent cells can be eliminated by targeting their ability to evade apoptosis, which can be achieved by upregulating pro-apoptotic proteins. ABT-263, an orally bioavailable inhibitor of Bcl-2 family members, initiates apoptosis as early as 2 hours after administration in preclinical models. However, drug concentration shows large variability, and cell lines differ markedly in sensitivity, ranging from highly sensitive to resistant [36]. In Cal33 cells, the high baseline of Bcl-xL expression likely explains the early response to ABT-263 monotherapy. Other studies have similarly reported Bcl-xL upregulation in HNSCC, correlating with impaired apoptosis and reduced therapy response [37, 38].

In our experiments, we applied the drug concurrently with irradiation at concentrations that produced minimal effects on their own but showed significant synergy when combined with radiation. This suggests that dose reduction may help limit side effects. In combination with radiotherapy, we observed synergistic effects on late apoptosis/necrosis. While Bcl-xL was downregulated, Bax activation was pronounced in UPCI:SCC040 and present to a lesser extent in Cal33 cells. Additional Bcl-2-family proteins are likely involved and may influence the effects of ABT-263. Mcl1, for example, has been identified as an inhibitor of ABT-263-induced radiosensitization in colon, lung and head and neck cancers [39, 40], and its role in the differences observed between our two cell lines warrants further investigation.

The decline in pro-Caspase 9 indicates Caspase-dependent activation of apoptosis. Lamin B1 expression decreased after drug administration—an unexpected observation, as Lamin B1 loss is a hallmark of senescence. Nevertheless, flow-cytometric analysis confirmed the removal of senescent cells. Because Lamin B1 is a component of the nuclear membrane and the nuclear envelope is degraded during apoptosis by caspase activity, its reduction is consistent with apoptotic processes [21]. Conversely, Lukasova et al. reported that Lamin B1 degradation is necessary for irradiation-induced senescence [41], suggesting that treatment regime was insufficient to restore Lamin B1 levels.

Cal33 cells were more sensitive to ABT-263 than UPCI:SCC040 cells. We observed a profound radiosensitization with irradiation: viability decreased at 1 µM ABT-263, and clonogenic assays showed strong radiosensitization in Cal33 cells, consistent with previous findings with pan-Bcl-2 inhibitors in HNSCC [42]. In contrast, UPCI:SCC040 cells responded to ABT-263 only at 5 µM, and radiosensitization was not observed with clonogenic assays.

Tuomainen et al. reported synergistic effects of ABT-263 and irradiation on apoptosis and proliferation [43], which is in agreement with our observations. In our study, we further assessed clonogenic survival, a functional endpoint that more accurately reflects long-term reproductive capacity and therapeutic relevance. Notably, radiosensitization was observed exclusively in Cal33 cells. This finding is particularly relevant because UPCI:SCC040 cells represent a subset of HNSCC characterized by pronounced radioresistance and elevated SASP cytokine expression, rendering this phenotype especially difficult to target therapeutically. For this aggressive subtype, however, the combined treatment regimen conferred no detectable benefit. Additional screening of further cell lines representative of this phenotype will be necessary to substantiate these results.

We hypothesized that the striking differences in CXCR2 gene expression between the two cell lines could account for the variability in treatment response. CXCR2 expression increased in UPCI:SCC040, but decreased in Cal33 cells following treatment. Indeed, CXCR2 knockdown with siRNA enhanced the radiosensitizing effect of ABT-263 in UPCI:SCC040 cells. DNA damage foci analysis confirmed that radiosensitization by ABT-263 is not driven by additional DNA damage but rather by apoptosis induction and reduced CXCR2 signaling. Although knockdown did not achieve the same degree of radiosensitization observed in Cal33 cells, CXCR2 expression can now be considered a contributor to therapy resistance. The CXCR2 gene promotor contains two NF-${\rm{\kappa }}$B binding sites which may contribute to its irradiation-induced regulation. In addition, posttranscriptional regulation by miRNAs, for example miR-940 was described in tongue squamous cell carcinoma [44, 45]. CXCR2 is a G-protein coupled receptor with pro-tumorigenic functions, including activation of MAPK signaling, proliferation, NF-${\rm{\kappa }}$B activation, and migration [44]. It shares high homology with CXCR1, and both receptors bind CXCL8 [46]. However, tumor cells vary widely in CXCR2 expression and ligand binding because of differences in genetics, epigenetics, microenvironmental signals, and selective pressures during tumor evolution. These aspects were not addressed in the present study and therefore limit the generalizability of our conclusions, as we analyzed only two cell lines, albeit with contrasting CXCR2 expression profiles.

The decision to focus on Cal33 and UPCI:SCC040 was based on the premise that only aggressive, radioresistant HNSCC cell lines exhibiting high levels of radiation-induced senescence and a pronounced senescence-associated secretory phenotype (SASP) are likely to benefit from the applied treatment strategy [13, 14]. Other commonly used HNSCC cell lines, such as UM-SCC-11B or UM-SCC-22B, also display variability in CXCR2 expression [47]; however, these models show only low levels of senescence induction following irradiation [48]. In contrast, UM-SCC-14B exhibits robust radiation-induced senescence [48] and, notably, increased CXCR2 expression when cultured under stem-like conditions [49]. This characteristic may render UM-SCC-14B a valuable model for future studies aimed at validating and extending the findings of the present work.

Our findings indicate that CXCR2 expression supports therapy resistance. After irradiation, the cell lines secrete high levels of CXCR2-targeting factors. ABT-263 only partially reduced irradiation-induced expression of the ligands IL8, CXCL1, CXCL2, CXCL5 and CXCL7, and IL8 and CXCL1 protein secretion remained significantly elevated compared with controls. This positions CXCR2 as a central mediator that must be inactivated or shielded from ligand binding. Comparable conclusions were drawn in the study by Schoetz et al. [14], where Metformin, a potent NF-${\rm{\kappa }}$B inhibitor, achieved radiosensitization when IL8 and CXCL1 chemokine production were suppressed.

Beyond tumor-intrinsic effects, secretion of these factors into the tumor microenvironment influences other cellular components. A recent preclinical study in murine colorectal and breast tumors [50] showed that non-homogenous irradiation increased CXCR2-binding ligands, with upregulation localized to high-dose regions. Although therapy-induced senescence was not addressed, the authors identified infiltration of immunosuppressive neutrophils—highly expressing CXCR2 and attracted by its ligands—as a key mechanism. In this context, CXCR2 inhibition markedly improved immunoradiotherapy. Another study found that CXCR1/2 inhibition sensitized HPV-negative HNSCC models to docetaxel both intrinsically and through immune-related effects [47]. While senescence was not the focus of that work, docetaxel is a known senescence inducer. Together, these studies highlight the importance of targeting senescence-associated signaling and the SASP, which are common consequences of radiotherapy.

## Conclusions

**Fig. 7:**
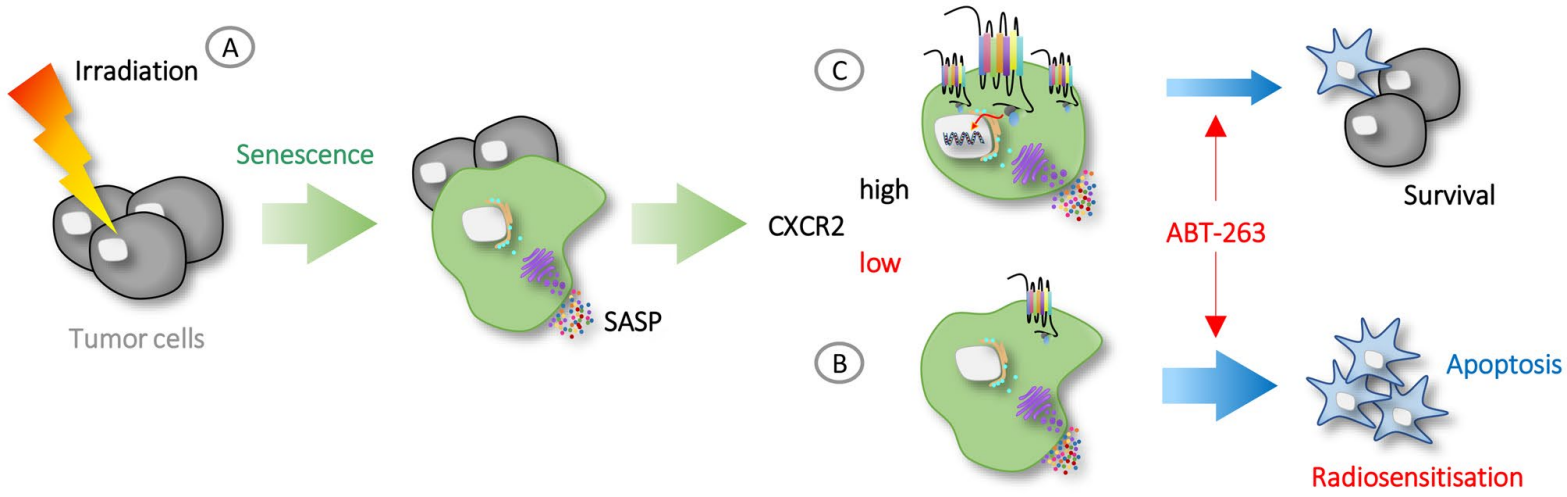
Conclusion. (**A**) Irradiation induces premature senescence in tumor cells and triggers SASP secretion. (**B**) In cells with reduced CXCR2 expression, treatment with the senolytic ABT-263 enhances intrinsic apoptosis and clearance of senescent cells, leading to radiosensitization. (**C**) In cells where CXCR2 expression increases after irradiation, ABT-263 induces limited apoptosis, allowing senescent cells to persist and support survival

Senescence is an unavoidable consequence of radiotherapy, and corresponding molecular signatures are detectable in the peripheral blood of tumor patients undergoing treatment [51]. Targeting senescent cells represents an effective strategy for radiosensitization in HNSCC [52, 53], with accumulating evidence implicating SASP expression and CXCR2 signaling as key mediators of this effect [54–56]. Our findings provide a rationale for the use of senolytics in combination with irradiation and identify CXCR2 as a key component regulating therapy success (Fig. 7). Further investigation of this signaling axis in HNSCC tumor cells and within the tumor microenvironment of patient samples is warranted to clarify the potential of CXCR2 as a druggable target in the development of improved therapies.

## Electronic supplementary material

Below is the link to the electronic supplementary material.

Supplementary material 1

Supplementary material 2

Supplementary material 3

## Data Availability

The data generated in this study are available upon reasonable request from the corresponding author.

## Abbreviations

C12FDG: 5-dodecanoylaminofluorescein-di-β-D-galactopyranoside
CI: Combination index
D10: Dose at 10% survival
DDR: DNA damage response
DSB: Double-strand break
HNSCC: Head and neck squamous cell carcinoma
HSA: Highest Single Agent
PI: propidiumiodide
SA-β-gal: Senescence-associated β-galactosidase
SASP: Senescence-associated secretory phenotype
SEM: Standard error of the mean
siCXCR2: CXCR2 sequence-specific siRNA
siNonT: Non-targeting control siRNA
STR: Short tandem repeat
