# Supplementary material for: CXCR2 affects sensitization of radioresistant HPV-negative head and neck squamous cell carcinoma cells by ABT-263
Source: Radiat Oncol. 2026 Feb 12;21:33. doi: 10.1186/s13014-026-02798-w (PMC12922328; doi:10.1186/s13014-026-02798-w)
Supplement: Supplementary file 1 — Supplementary material 1 [file 13014_2026_2798_MOESM1_ESM.pdf]

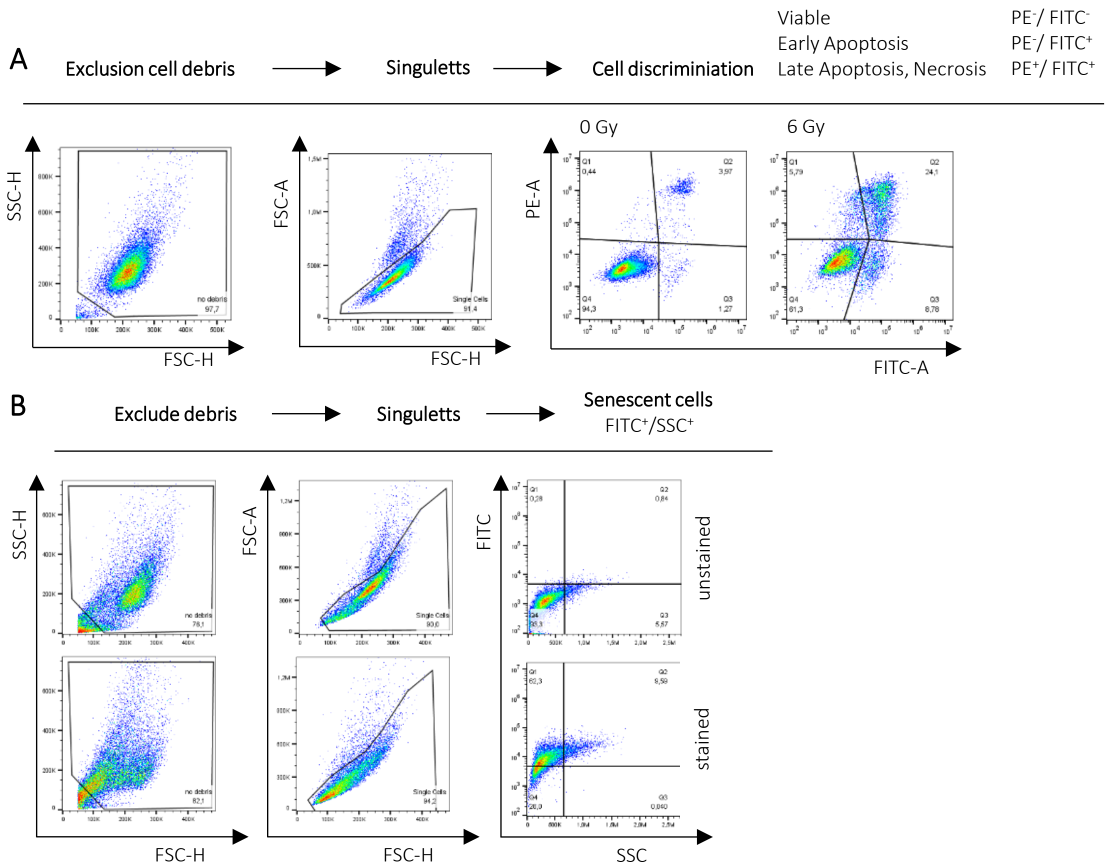

Supplementary Figure 1. Gating strategy for (A) Annexin V assay and (B) senescence assay flow cytometry. (A) Cell debris was excluded by gating the cells in forward scatter and side scatter (height). Singuletts were discriminated by gating the single cells in forward scatter area and height. DNA content of dead perforated cells was stained by propidium iodide and external lipopolysaccharide of membrane-intact cells was stained with AnnexinV-FITC. Viable cells were determined as being negative for PE and FITC, whereas cells in early apoptosis appear PE-negative and FITC-positive. Necrotic cells stain for both, PE and FITC. (B) Cell debris was excluded by gating the cells in forward scatterr and side scatter. Singuletts were discriminated by gating the single cells in forward scatter area and height. Senescent cells were identified based on their senescence-associated β-galactosidase (SA-β-Gal) activity, which causes the substrate conversion of C12FDG into a strong C12 fluorescein signal ($FITC^+$) and exhibiting high granularity (SSC^+).

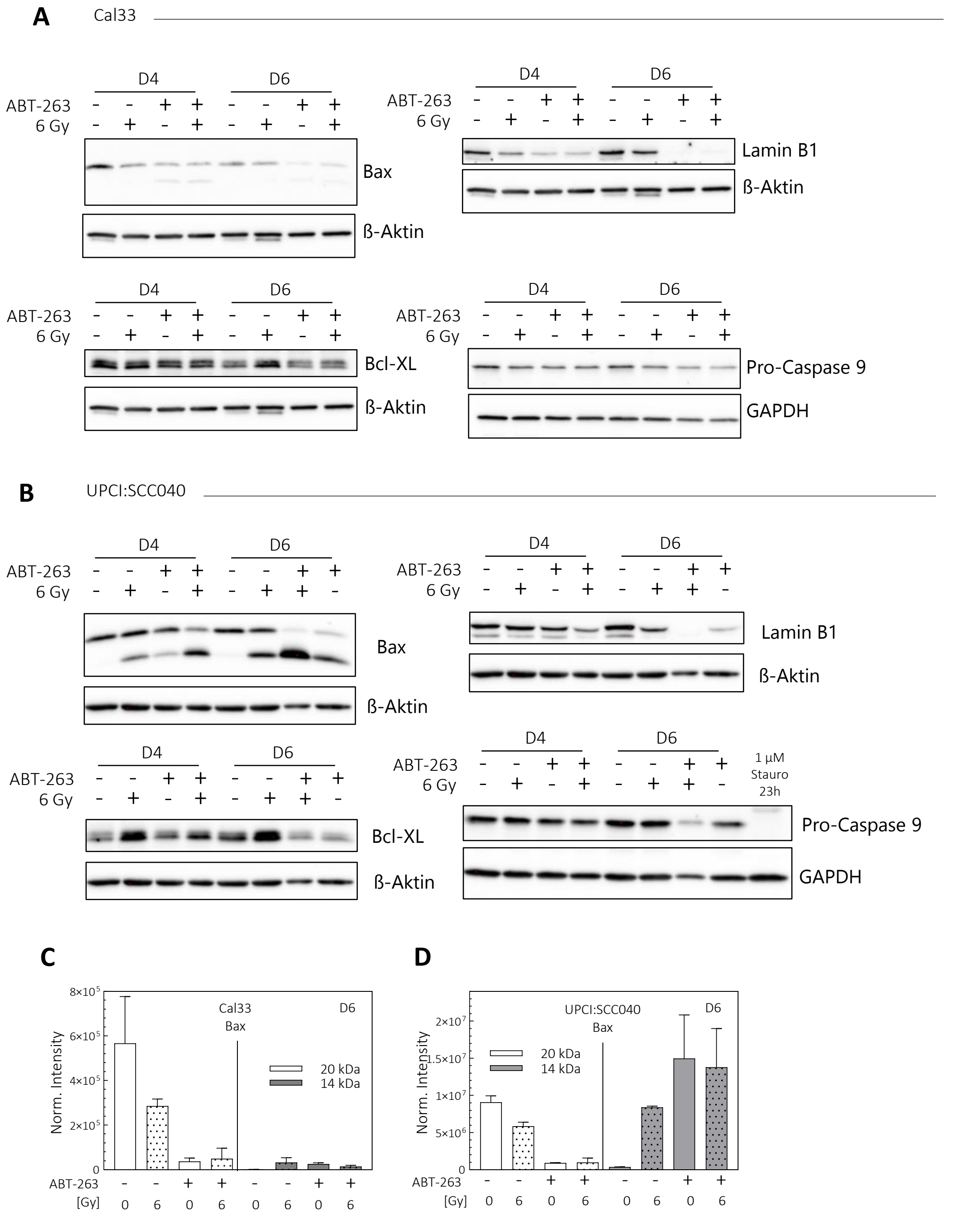

Supplementary Figure 2. Western Blots for data analysis in figure 1 and 2. A) Cal33 B) UPCI:SCC040. C) D6 quantification protein Bax. Representative Western Blots and graphic analysis of densitometry of N=2 replicates are displayed for Bax D6. Values are shown as means +/- SEM .

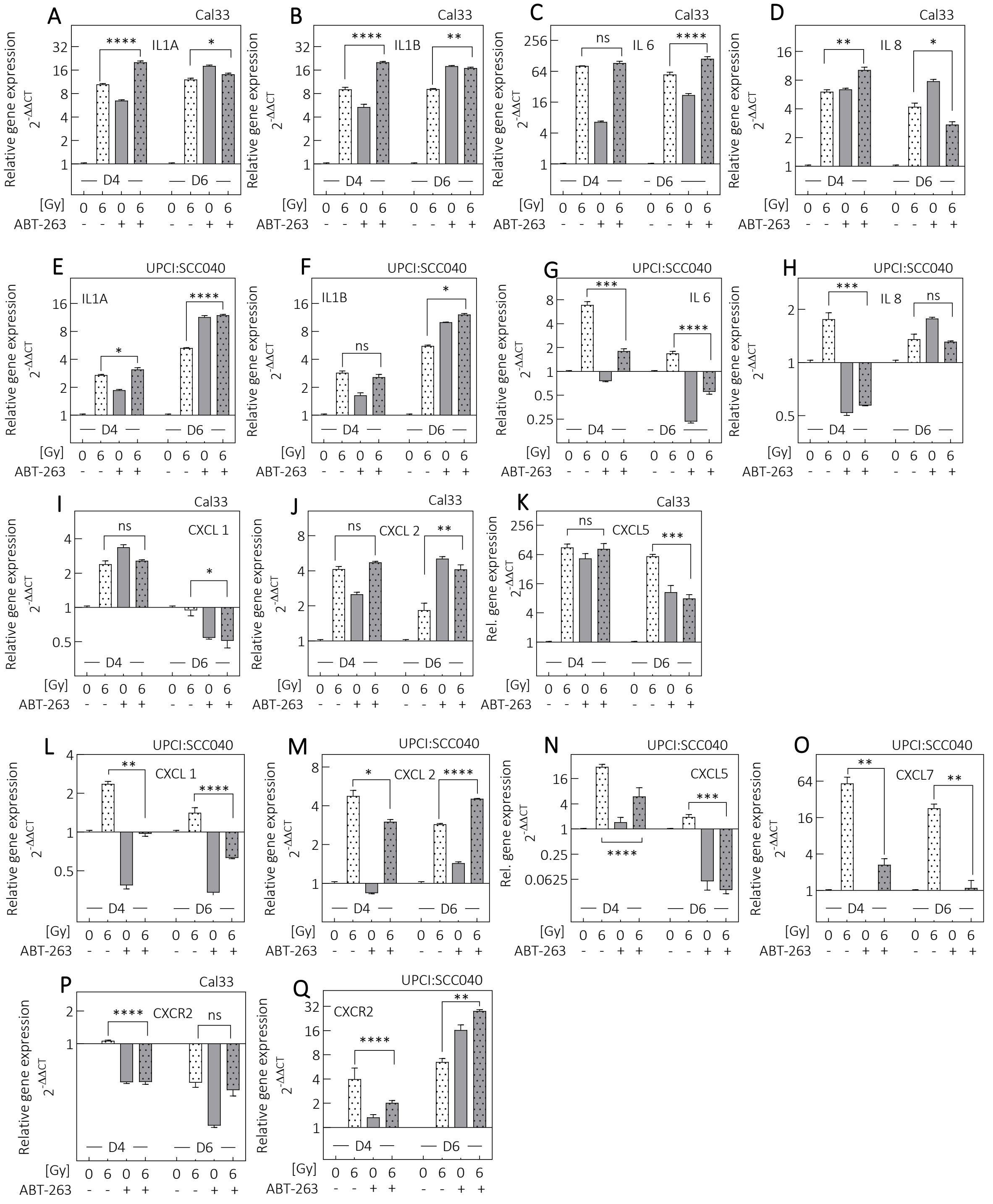

Supplementary Figure 3. Relative gene expression ($2^{-\Delta\Delta CT}$) of various secreted factors as determined by qRTPCR in Cal33 (A-D,I-K,P) and UPCI:SCC040 cells (E-H,L-O,Q). Cells were treated concomitantly with 1 µM ABT-263 for Cal33 and 5 µM ABT-263 for UPCI:SCC040 cells and 6 Gy irradiation followed by analysis on day 4 (D4) and day 6 (D6) after treatment. Values are shown as means +/- SEM of N = 2 replicates. A Student´s T test was performed to determine significance levels $p < 0.05$ (*), $p < 0.01$ (**), $p < 0.001$ (***), $p < 0.0001$ (****), ns: not significant .

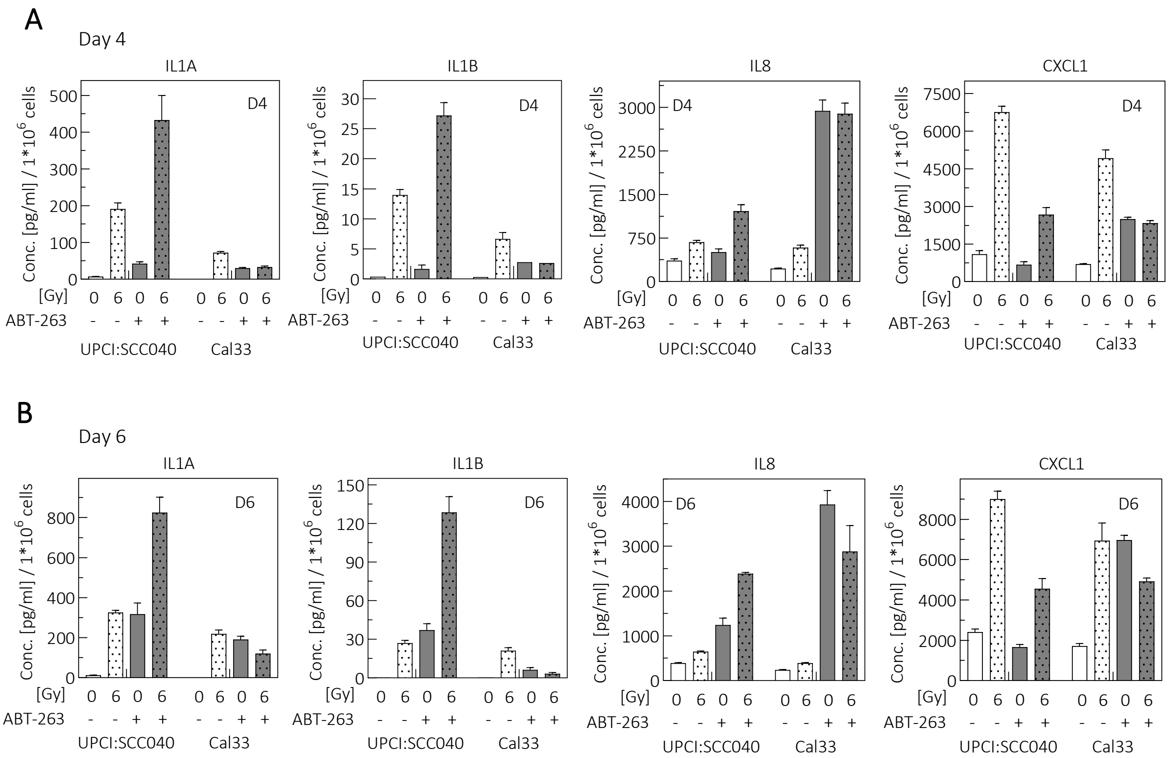

Supplementary Figure 4. Detection of the secreted proteins IL1A, IL1B, IL8 and CXCL1 by ELISA on D4 (A) and D6 (B) after treatment with 1/5 µM ABT-263 and 6 Gy irradiation. Concentration of protein in pg/ml was calibrated to the cell number in the corresponding sample. Values are shown as means +/- SEM of N ≥ 3 replicates.
