# Supplementary material for: CXCR2 affects sensitization of radioresistant HPV-negative head and neck squamous cell carcinoma cells by ABT-263
Source: Radiat Oncol. 2026 Feb 12;21:33. doi: 10.1186/s13014-026-02798-w (PMC12922328; doi:10.1186/s13014-026-02798-w)

Supplementary File – Unprocessed Western Blots

Cal33 — Fig.1 C & Fig.2 C

Replicate 1 Gel 1

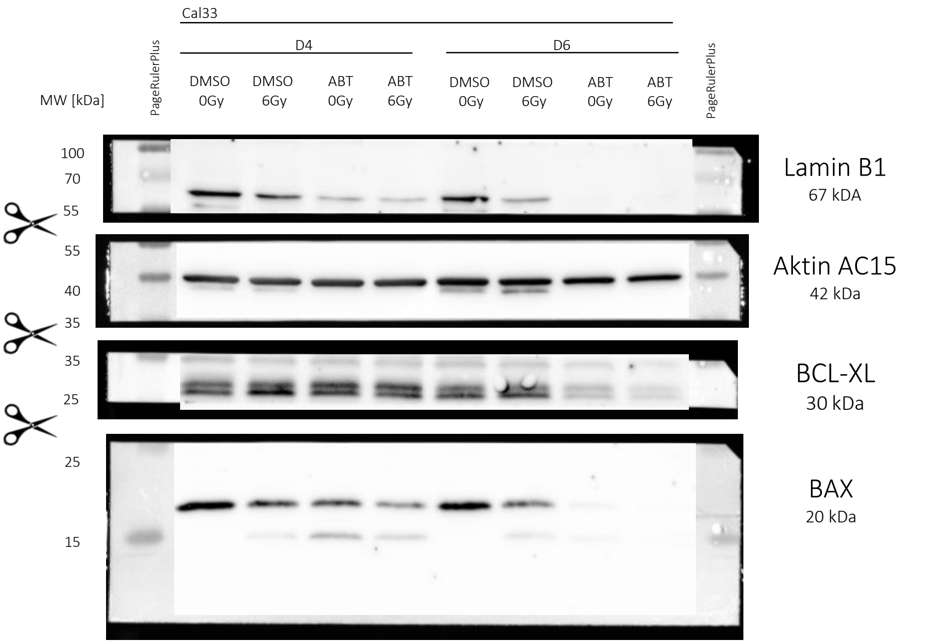

Note
This blot was cut into 4 pieces after transfer to the membrane and before antibody incubation.

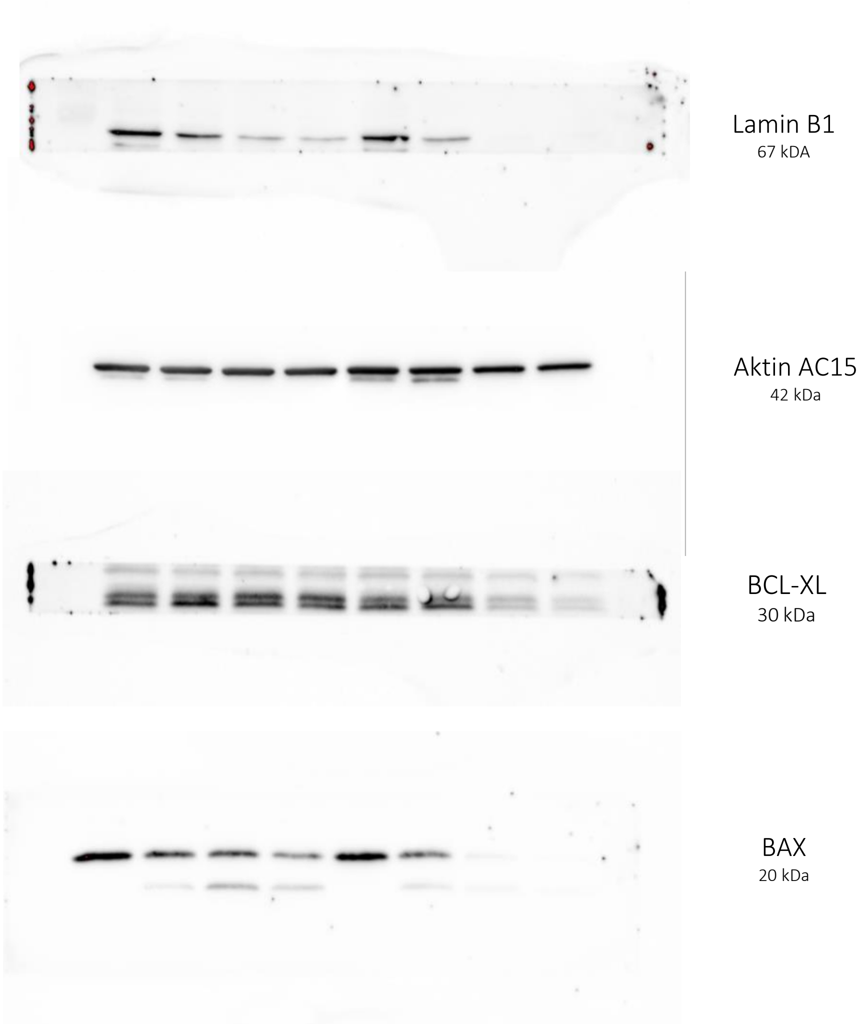

Cal33 — Fig.1 C & Fig.2 C

Replicate 1 Gel 2

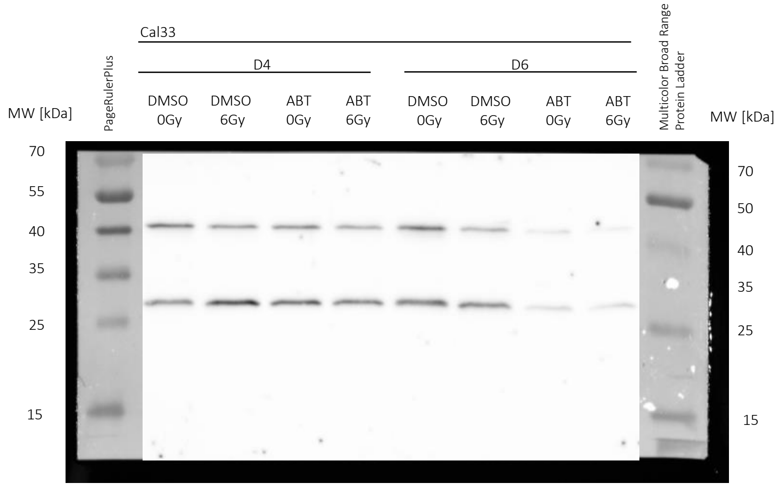

Casp9 #9502

Casp9

35/37/45 kDa

Note

This blot was first stained with the antibody against procaspase 9, then all antibodies were removed by stripping, and in a second step, the GAPDH signal was determined.

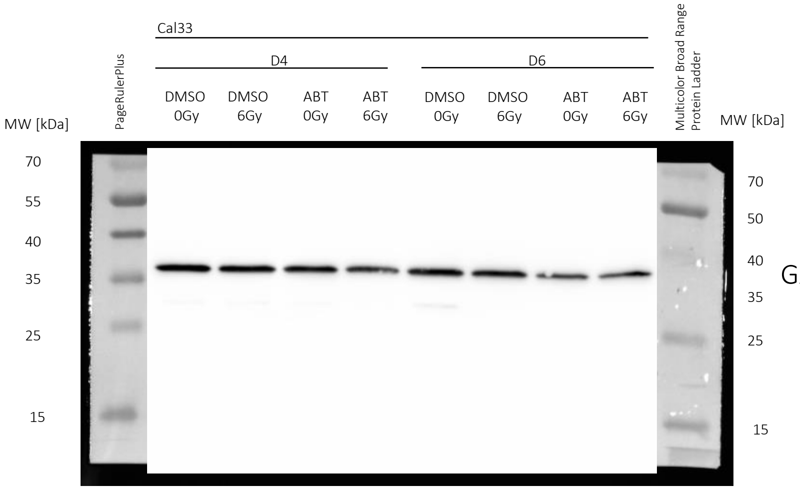

GAPDH (14C10) #2118

HK 37 kDa

Casp9

35/37/45 kDa

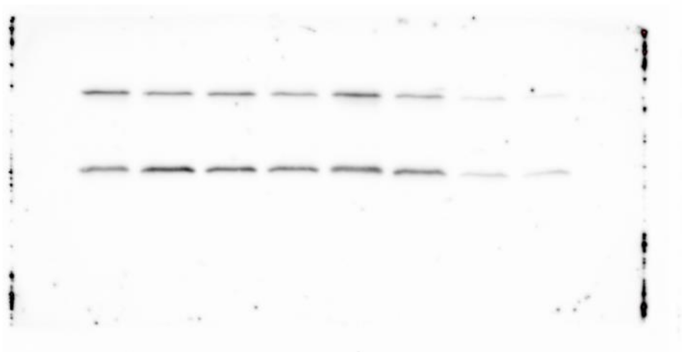

GAPDH (14C10) #2118

HK 37 kDa

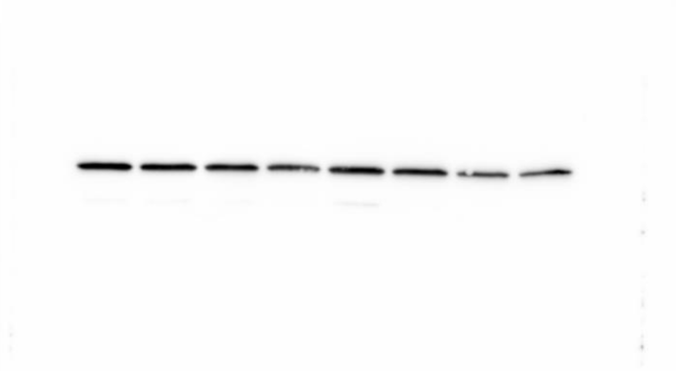

Cal33 Supp. Fig.2 A

Replicate 2 Gel 1

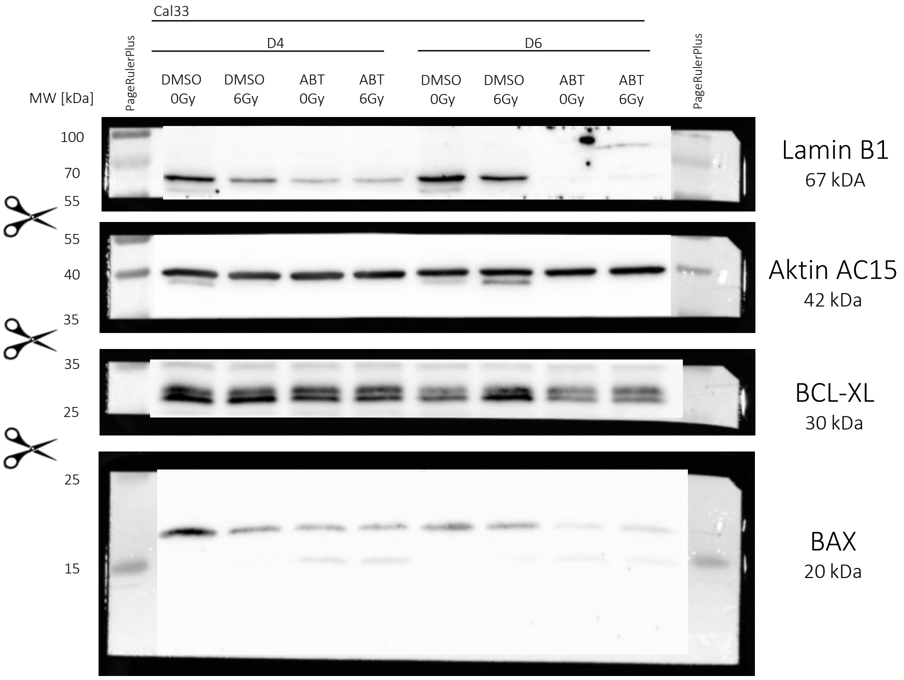

Note

This blot was cut into 4 pieces after transfer to the membrane and before antibody incubation.

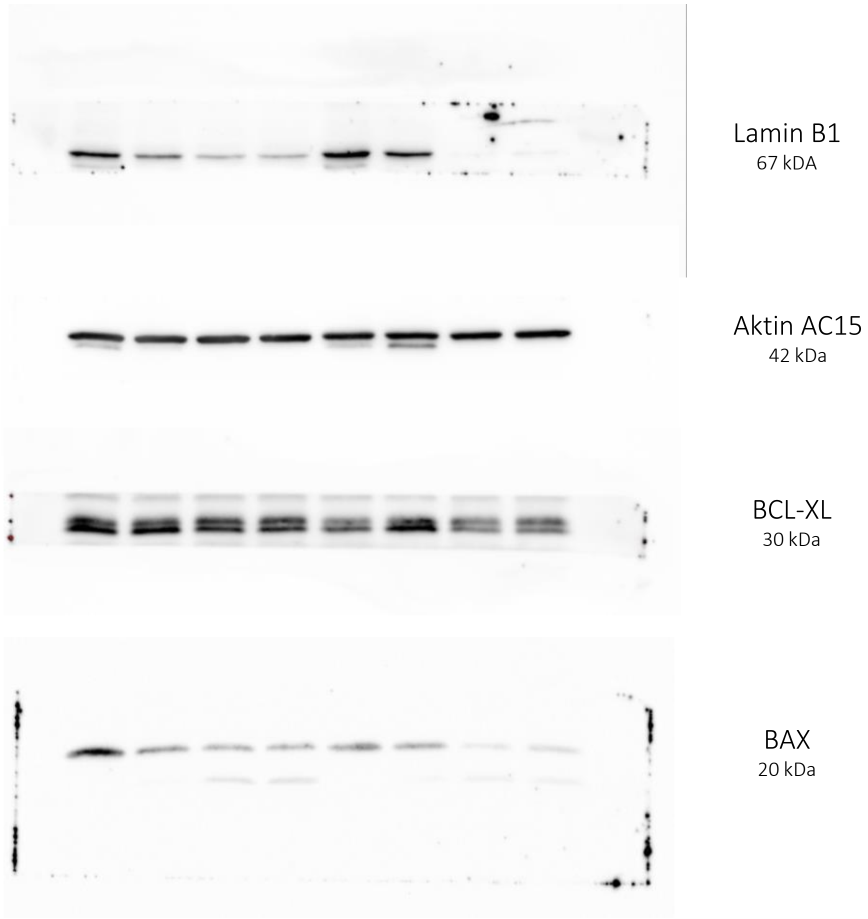

Cal33 — Supp. Fig.2 A

Replicate 2 Gel 2

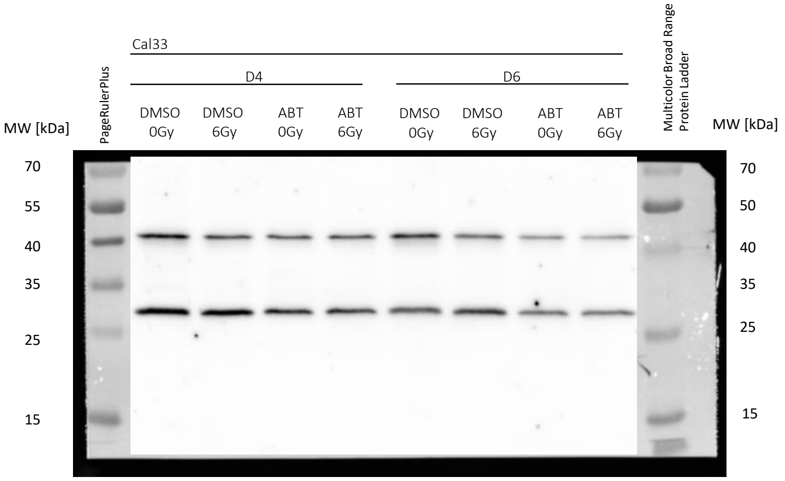

Casp9 #9502
Casp9
35/37/45 kDa

Note
This blot was first stained with the antibody against procaspase 9, then all antibodies were removed by stripping, and in a second step, the GAPDH signal was determined.

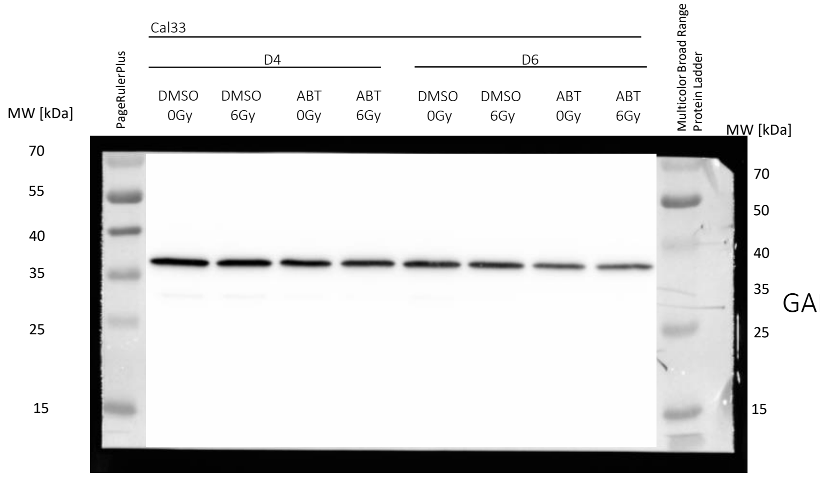

GAPDH (14C10) #2118
HK 37 kDa

Casp9
35/37/45 kDa

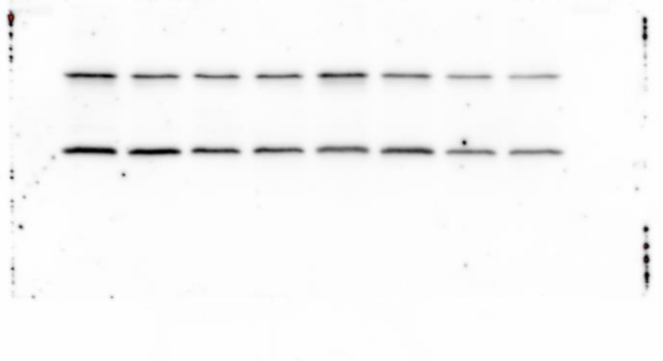

GAPDH (14C10) #2118
HK 37 kDa

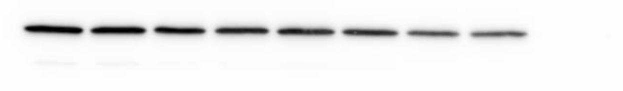

UPCI:SCC040 Supp. Fig.2 B

Replicate 1 Gel 1

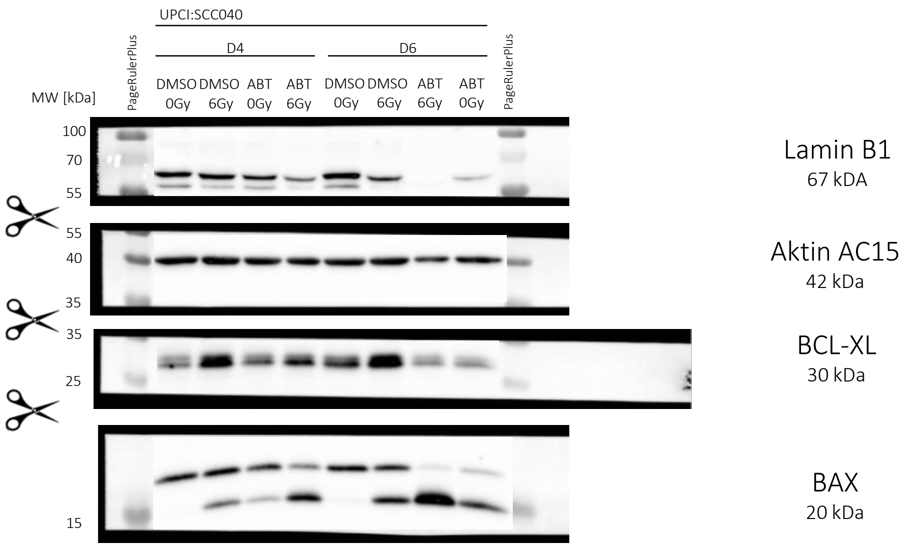

Note
This blot was cut into 4 pieces after transfer to the membrane and before antibody incubation.

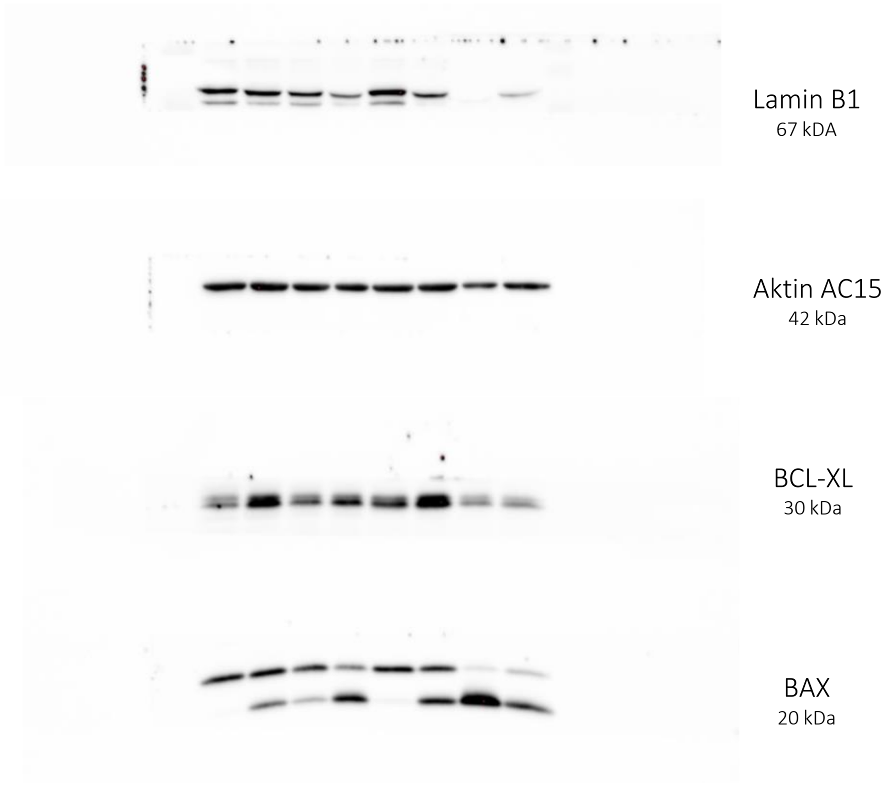

UPCI:SCC040 — Supp. Fig.2 B

Replicate 1 Gel 2

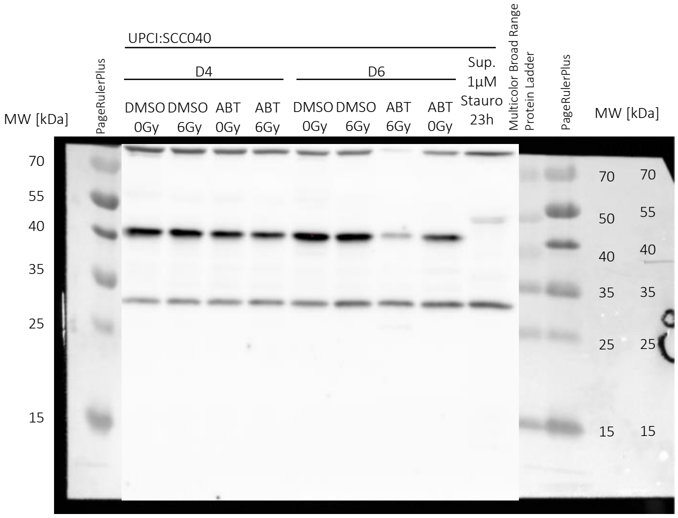

Casp9 #9502
Casp9
35/37/45 kDa

Note
This blot was first stained with the antibody against procaspase 9, then all antibodies were removed by stripping, and in a second step, the GAPDH signal was determined.

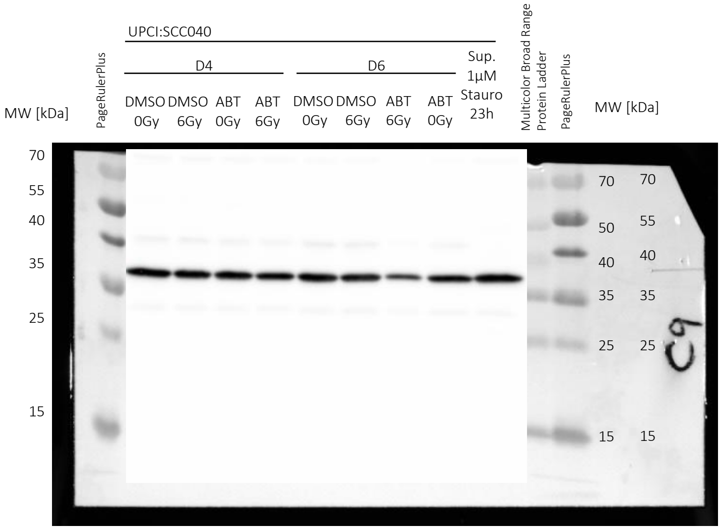

GAPDH (14C10) #2118
HK 37 kDa

Casp9
35/37/45 kDa

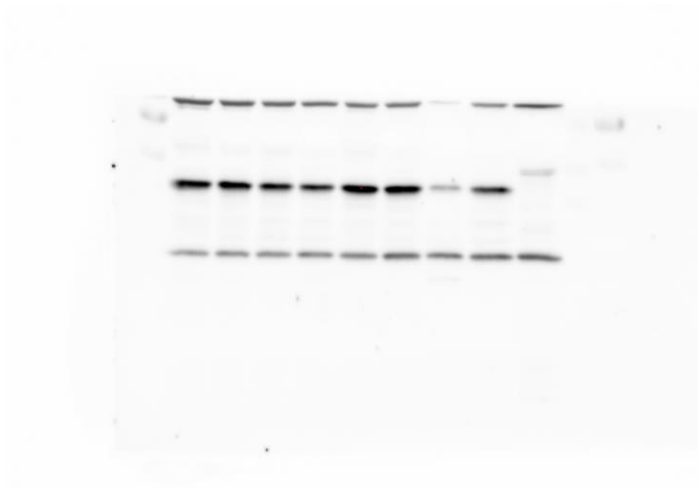

GAPDH (14C10) #2118
HK 37 kDa

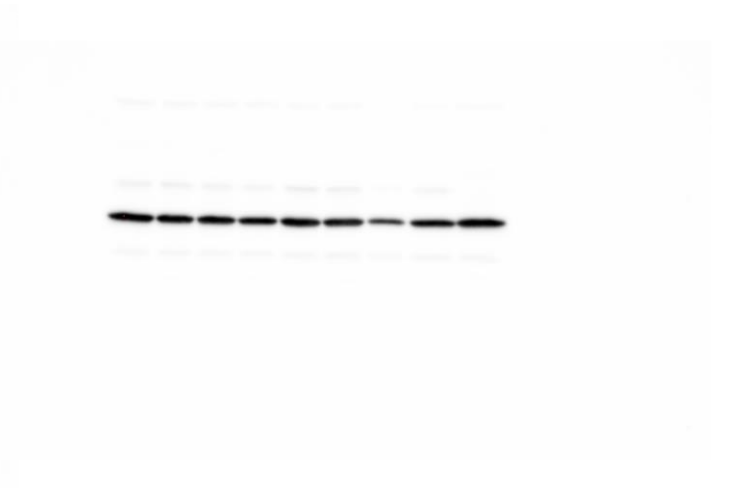

UPCI:SCC040 — Fig.1 D & Fig.2 D

Replicate 2 Gel 1

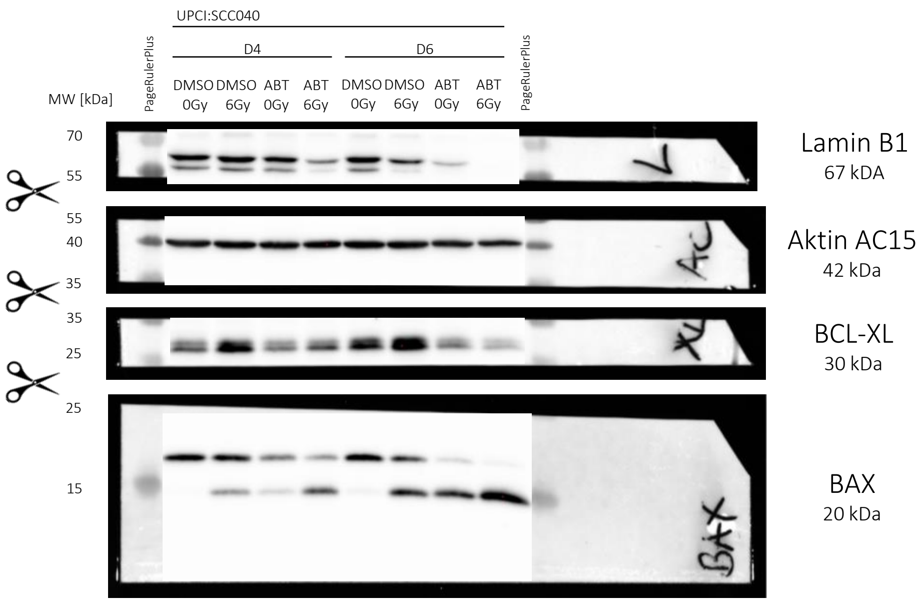

Note

This blot was cut into 4 pieces after transfer to the membrane and before antibody incubation.

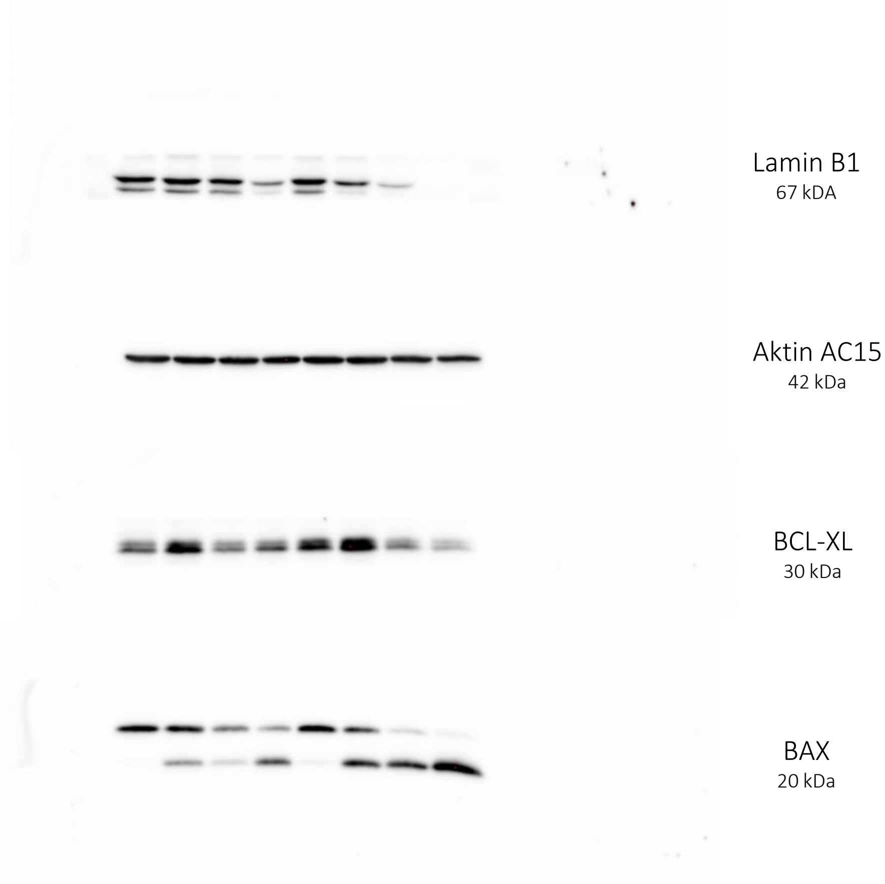

UPCI:SCC040 — Fig.1 D & Fig.2 D

Replicate 2 Gel 2

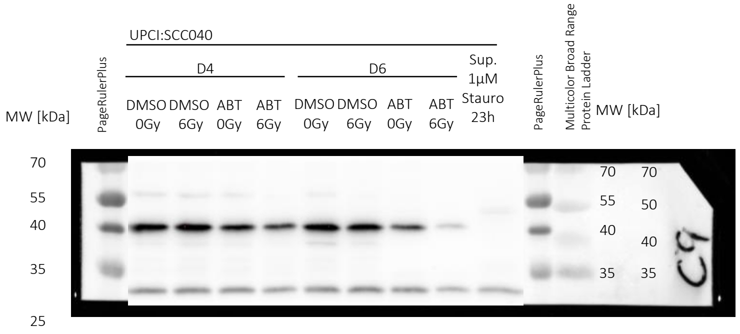

Casp9 #9502
Casp9
35/37/45 kDa

Note
This blot was first stained with the antibody against procaspase 9, then all antibodies were removed by stripping, and in a second step, the GAPDH signal was determined.

In contrast to replicate 1, the blot was cut at the 25 kDa marker band.

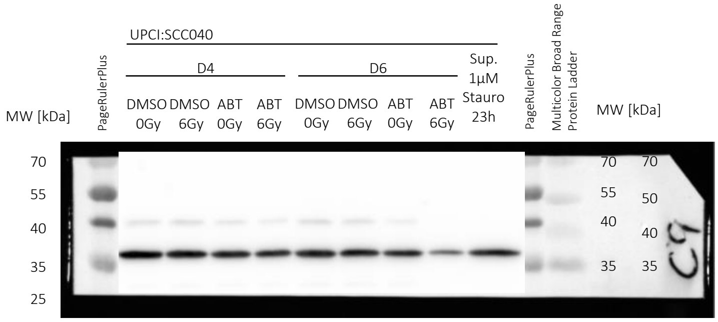

GAPDH (14C10) #2118
HK 37 kDa

Casp9
35/37/45 kDa

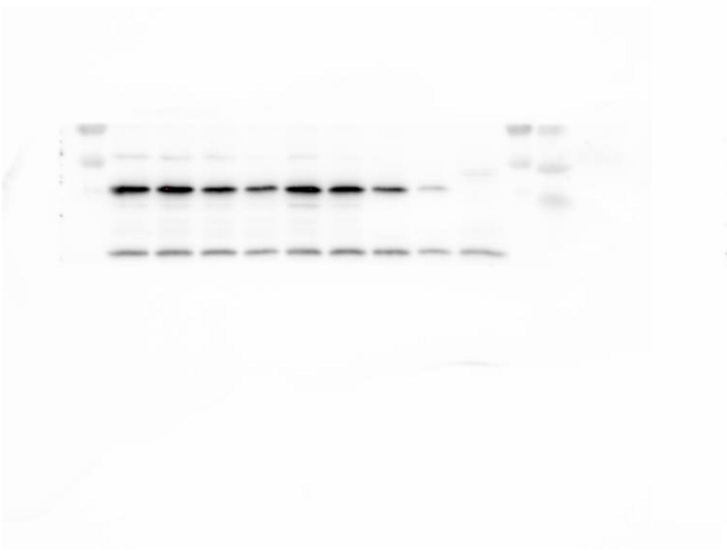

GAPDH (14C10) #2118

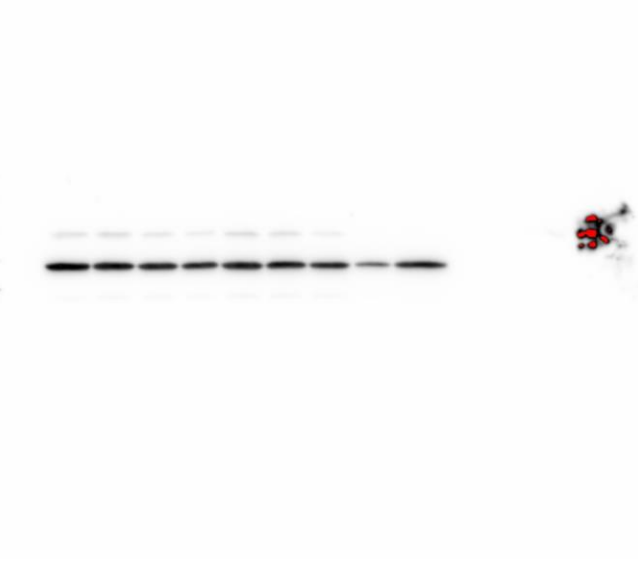

Supplement: Supplementary file 2 — Supplementary material 2 [file 13014_2026_2798_MOESM2_ESM.pdf]
